# The Global S$_1$ Tide in Earth’s Nutation

**DOI:** 10.1007/s10712-016-9365-3

**Published:** 2016-02-15

**Authors:** Michael Schindelegger, David Einšpigel, David Salstein, Johannes Böhm

**Affiliations:** Department of Geodesy and Geoinformation, TU Wien, Gußhausstraße 27–29, 1040 Vienna, Austria; School of Cosmic Physics, Dublin Institute for Advanced Studies, Dublin, Ireland; Department of Geophysics, Charles University in Prague, Prague, Czech Republic; Atmospheric and Environmental Research, Inc., Lexington, MA USA

**Keywords:** Earth rotation variations, Nutation, Geophysical excitation, Atmospheric tides, Ocean tides

## Abstract

Diurnal S$_1$ tidal oscillations in the coupled atmosphere–ocean system induce small perturbations of Earth’s prograde annual nutation, but matching geophysical model estimates of this Sun-synchronous rotation signal with the observed effect in geodetic Very Long Baseline Interferometry (VLBI) data has thus far been elusive. The present study assesses the problem from a geophysical model perspective, using four modern-day atmospheric assimilation systems and a consistently forced barotropic ocean model that dissipates its energy excess in the global abyssal ocean through a parameterized tidal conversion scheme. The use of contemporary meteorological data does, however, not guarantee accurate nutation estimates per se; two of the probed datasets produce atmosphere–ocean-driven S$_1$ terms that deviate by more than 30 $\upmu$as (microarcseconds) from the VLBI-observed harmonic of $-16.2+i113.4$ $\upmu$as. Partial deficiencies of these models in the diurnal band are also borne out by a validation of the air pressure tide against barometric in situ estimates as well as comparisons of simulated sea surface elevations with a global network of S$_1$ tide gauge determinations. Credence is lent to the global S$_1$ tide derived from the Modern-Era Retrospective Analysis for Research and Applications (MERRA) and the operational model of the European Centre for Medium-Range Weather Forecasts (ECMWF). When averaged over a temporal range of 2004 to 2013, their nutation contributions are estimated to be $-8.0+i106.0$ $\upmu$as (MERRA) and $-9.4+i121.8$ $\upmu$as (ECMWF operational), thus being virtually equivalent with the VLBI estimate. This remarkably close agreement will likely aid forthcoming nutation theories in their unambiguous a priori account of Earth’s prograde annual celestial motion.

## Introduction

Describing variations of our planet’s orientation in space is a multidisciplinary subject matter that has occupied the attention of mathematicians, astronomers, and geophysicists alike. Nutations, that is, periodic motions of a predefined physical or conventional reference axis with respect to an inertial system, have been classically modeled in a Lagrangian or Hamiltonian framework (Woolard 1953; Kinoshita 1977) as the rigid Earth response to gravitational lunisolar torques. The estimates of these standard treatments are accurate to a few tenths of mas (milliarcseconds), but the advent of precise observational data as well as the pursuit of insights into Earth’s internal constitution has stimulated the development of non-rigid nutation theories for a realistic Earth (Jeffreys and Vicente 1957; Molodensky 1961; Sasao et al. 1980). In 1980, the International Astronomical Union (IAU) adopted theoretical values of the lunisolar forced nutations for an elliptical, oceanless, elastic Earth with a fluid outer core and solid inner core (Wahr 1981), though the geophysical approximations and omissions within that model were soon to become larger than the requirements posed by space geodetic techniques such as VLBI (Very Long Baseline Interferometry). A timely formation of a new nutation series, which has been the reference model since its endorsement by the IAU in 2000, is documented in Mathews et al. (2002) (MHB for short) and explicitly allows for mantle anelasticity, inner core dynamics, and non-hydrostatic equilibrium effects. Basic Earth parameters that govern the nutation response to lunisolar and planetary torques are constrained to their “best estimates” from a least-squares fit of the theoretical nutation expressions to VLBI results. This semi-analytical approach to modeling Earth’s nutation leaves residuals with observational data below 0.1 mas.

Some effort has been devoted by MHB to properly account for the nutation perturbations associated with the Earth’s fluid layers. These contributions range from a few tens of $\upmu$as (microarcseconds) to 1 mas in amplitude and can be understood as the manifestations of (quasi-)diurnal atmosphere–ocean dynamics in the terrestrial frame. Their underlying excitation mechanisms are twofold, comprising (1) the daily cycle of solar heating and (2) the differential gravitational forces that directly act upon the atmosphere and ocean and produce global-scale waves known as tides. At the major diurnal tidal frequencies, the oceanic variability is almost exclusively driven by the gravitational influence with minute modulations related to the hydrodynamic response to atmospheric forcing. Satellite altimetry provides an accurate global record of these signals in the modern ocean and is also typically used to infer the associated oceanic angular momentum (OAM) variations of the largest diurnal tides, K$_1$, P$_1$, O$_1$, and Q$_1$. Early OAM determinations for these four waves (Chao et al. 1996) were adopted by MHB to predict the full OAM spectrum across the diurnal band and subsequently correct the equations of motion and anelasticity in the nutation theory.

Tides in the atmosphere around the central diurnal period of S$_1$ are capable of exciting small additional, seasonal nutation waves that exceed the statistical uncertainties of VLBI-based parameters on the order of 10 $\upmu$as (Dehant et al. 2003). The gravitational components of these oscillations are—to the extent they have not been implicitly accounted for in the MHB model—negligibly small (Bizouard and Lambert 2002) and in fact overshadowed by thermal tides due to periodic radiation and absorption processes (Chapman and Lindzen 1970). Nutation contributions of some minor radiational constituents, such as the P$_1$ tide, have been thoroughly addressed by MHB, yet the main S$_1$ wave proved to pose some sort of conundrum to the authors. Whereas the VLBI data (1979.8–1999.11) distinctly testified to the existence of an S$_1$ influence in the form of a prograde annual nutation residual, available geophysical model estimates (Bizouard et al. 1998; Yseboodt et al. 2002) were deemed unreliable, and thus no “theoretical” account of the effect was incorporated into the dynamical equations. To compensate for this mismatch, MHB subtracted from the VLBI spectrum an a priori S$_1$ harmonic of somewhat more than 100 $\upmu$as and superimposed the very same signal as a post-fit correction term to the final nutation series.

**Fig. 1:**
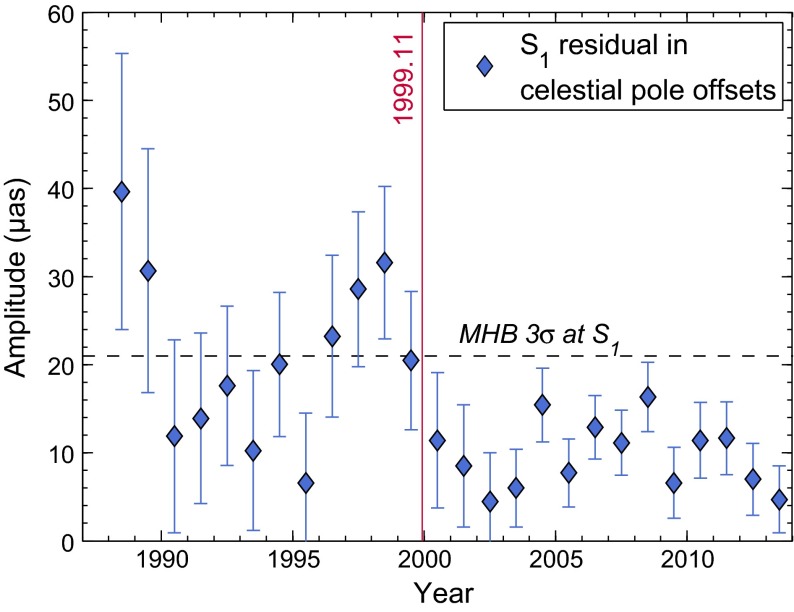
Prograde annual signal amplitude in the IAU2000 celestial pole offsets (CPO) w.r.t. the MHB model. Estimates are 3-year sliding window fits, and error bars indicate standard deviation (SD) in amplitude that have been propagated rigorously from the CPO errors. The MHB VLBI analysis, documented in Herring et al. (2002), involves data up to November 1999 (*red line*) and features a threefold SD of 21 $\upmu$as in the prograde annual band (*dashed black line*)

From a practical point of view, this approach is legitimate and in fact aided by the pronounced harmonic character of the unexplained nutation variability in the prograde annual band. Corrections to the MHB model, published as daily celestial pole offsets by the International Earth Rotation and Reference Systems Service (IERS), are generally below 20 $\upmu$as in the S$_1$ band, also for more recent years not included in the original MHB analysis; cf. Fig. 1. However, in the spirit of a theory that should be ultimately free of empirical adjustments (Fedorov et al. 1980) and also unambiguous in its account of the various effects at the prograde annual frequency, it is still worthwhile to strive for an independent S$_1$ estimate from geophysical fluid models.

Studies of this subject matter are required to accommodate not only the atmospheric portion of the tide but also the substantial 24-h oceanic mass redistributions driven by S$_1(p)$, the diurnal pressure variations at the sea surface. Hence, both atmospheric and oceanic oscillations are closely interrelated aspects of the same “global S$_1$ tide,” labeled as such by Ray and Egbert (2004) as well as in the context of the present work. Owing to its radiational origin, the oceanic S$_1$ variability might be also perceived as an indirect influence of the atmosphere on the rotation of the solid Earth, and it has therefore been occasionally classified as a “non-tidal” phenomenon (Brzeziński et al. 2004; de Viron et al. 2004)—a terminology that shall, however, not be used in the following.

Investigations of the S$_1$ effect in nutation on the basis of dynamically coupled atmosphere–ocean models have been pursued primarily by A. Brzeziński and collaborators; cf. Brzeziński et al. (2004), Brzeziński (2011) and references therein. These studies employed different atmospheric analyses and ocean models both in a full 3D baroclinic formulation as well as 2D (constant density) barotropic versions that efficiently capture short-period hydrodynamic processes. The inferred atmosphere–ocean excitation terms of the prograde annual nutation vary substantially, though, both among each other and from the geodetic VLBI value, with deviations usually being larger than 50 $\upmu$as. While the VLBI estimate itself is possibly perturbed by other, imperfectly modeled seasonal effects, we must proceed on the assumption that the probed general circulation models were not appropriately designed for S$_1$-related investigations. Another though brief assessment of the radiational tidal influence on nutation is given in de Viron et al. (2004) and their seemingly close agreement (<15 μas) with the VLBI value has been acknowledged by Dehant and Mathews (2009) (Sect. 10.11, *ibid.*). We think, however, that the results of de Viron et al. (2004) are questionable and actually affected by an incorrect conversion of excitation values to periodic nutation terms. This deficiency is particularly evident for their tabulated atmospheric contributions (P$_1$, S$_1$, and $\psi _1$), which are inconsistent with what has been documented for the very same atmospheric dataset by Koot and de Viron (2011) and Bizouard et al. (1998), even if one makes allowance for differences in the utilized transfer functions and the analyzed time spans. Note, e.g., that over the period 1991–2002, Fig. 2 of Koot and de Viron (2011) suggests $\sim$75 $\upmu$as for the S$_1$ out-of-phase term, while de Viron et al. (2004) specify a value of 38 $\upmu$as. Without further insight into the actual (corrected) S$_1$ nutation predictions of these authors, we will employ Brzeziński (2011) as a reference study by which our results can be measured.

Building on the elucidations of these pilot investigations, the key objective of the present work is to provide an up-to-date treatment of the global S$_1$ tidal effect in nutation and ultimately arrive at an explanation of MHB’s empirical prograde annual nutation term. The modern-day aspect of our effort resides in the use of four of the currently most advanced atmospheric assimilation systems, comprising three constant-model, retrospective analyses (so-called reanalyses) for a principal time span from 1994 to 2013, and a shorter, operational dataset (2004–2013) that stems from the near real-time weather analysis with a steadily improving model. This atmospheric portion of our study can be rightly understood as a continuation of similar earlier assessments (Bizouard et al. 1998; Yseboodt et al. 2002), and as such, it is partly motivated by the good agreement of atmospheric nutation estimates from reanalyses that are essentially the precursors of the presently tested models (Koot and de Viron 2011). To ensure conformity with these investigations, nutation values for the minor solar constituents ($\psi _1$, P$_1$, $\pi _1$, and $\phi _1$) are tabulated, even though our prime focus is on the S$_1$ tide throughout.

A second key theme of this work is to numerically model the dynamic ocean response to diurnal atmospheric pressure forcing, which has been a fruitful geophysical industry over the last decade; cf. Ray and Egbert (2004), Dobslaw and Thomas (2005), Ponte and Vinogradov (2007), or Carrère et al. (2012). Mere superpositions of these modeling results to our atmospheric excitation terms are invalid, though, and a rigorous treatment of the geophysically driven prograde annual nutation requires deducing hydrodynamic S$_1$ solutions and respective OAM values that are consistent with the utilized atmospheric datasets. We adopt the recent barotropic time-stepping model of Einšpigel and Martinec (2015), designated as DEBOT (Barotropic Ocean Tide model developed by D. Einšpigel), and implement the necessary modifications for the problem in hand. Specifically, to obtain S$_1$ tidal solutions that are on par with Ray and Egbert (2004), ocean self-attraction and loading effects (SAL, Ray 1998b) are accounted for in an iterative fashion and the overestimation of sea surface elevations in deep water is mitigated by a parameterized expression for the barotropic-to-baroclinic energy conversion over abyssal hills (Bell 1975; Jayne and St Laurent 2001). Moreover, forcing the same hydrodynamic model with different pressure tide solutions should go some way to reveal the dependence of the global/regional character of S$_1$ (and its OAM values) on variations in the barometric input data. This is a subtle issue that has yet not been addressed by the oceanographic community.

Our approach is a climatological one inasmuch as the present formulation of DEBOT only allows for a strictly harmonic pressure loading by the S$_1$ air tide, even though the temporal variability of this forcing can be large (Ray 1998a). Seasonal modulations of S$_1(p)$ by 1 cpy (cycle per year) correspond to the P$_1$ and K$_1$ constituents, inducing small radiational ocean tides that are automatically included in altimetric solutions of P$_1$ and K$_1$ and are thus of no practical significance. Variations on inter-annual timescales (e.g., Vial et al. 1994) pose, however, a more delicate challenge, which can be partly resolved by working with decadal-scale S$_1$ averages for the coupled atmosphere–ocean system. To determine a favorable, i.e., inter-annually “quiet” averaging period, we assess the S$_1$ variability both in the integrated atmospheric nutation values and in surface pressure. Specifically, the analysis of S$_1(p)$ is conceived as a validation of model pressure tides against “ground truth” estimates from 50 island- and buoy-based barometers. This comparison is in fact a vital (though limited) measure in deciphering the fine margins in quality among the different models regarding the diurnal cycle. Further observational constraints on our model-based investigations are supplied by S$_1$ determinations at 56 coastal tide gauges, which, to some extent, echo the varying degree of reliability of the simulated tidal heights from each atmospheric dataset.

The paper is organized as follows. Section 2 places the present and previous nutation studies in the context of an evolving collection of meteorological assimilation systems and describes the main characteristics of the four models utilized herein. Atmospheric excitation time series are computed and mapped to nutation amplitudes in Sect. 3, complemented by the validation of model pressure tides against in situ estimates from pelagic barometers. The ocean model and its hydrodynamic configuration are thoroughly discussed in Sect. 4, and we assess the quality of our forward simulations both from an angular momentum perspective as well as in a comparison to coastal tide gauges. Section 5 finally synthesizes atmospheric, oceanic, and VLBI nutation results to address the mismatch between theory and observation in the S$_1$ band.

## Meteorological Data for Nutation Studies

Solar tides in the atmosphere are not of immediate relevance to operational or retrospective analyses, yet a largely realistic model account of these oscillations is guaranteed by the use of insolational forcing physics in combination with in situ and remotely sensed meteorological data. Reanalyses, created by various weather agencies on the basis of an unchanging assimilation scheme over decades, are usually credited with a realistic long-term variability that also modulates the tides and, by implication, nutation amplitudes. Their products, issued with an invariable spatial and temporal resolution, have thus become the preferred means to investigate atmospheric effects in nutation. Table 1, taken from Schindelegger et al. (2015), summarizes some basic information of presently available reanalysis datasets, sorted by a rough generation index (Dee et al. 2015) that is thought to reflect the varying degree of sophistication in terms of model physics, resolution, and assimilation technique. Refer to the caption of Table 1 for any model abbreviations used in the following.

**Table 1:** Overview of current atmospheric reanalyses as operated by various meteorological agencies^a^

Name^b^	Source^b^	GI	Model vintage	Resolution (km)	Assimilation^c^
NCEP R1	NCEP	1	1995	210	3DVar
NCEP R2	NCEP	1	1995	210	3DVar
ERA-40	ECMWF	2	2001	125	3DVar
JRA-25	JMA	2	2002	120	3DVar
MERRA	NASA GMAO	3	2004	60	IAU
CFSR	NCEP	3	2004	40	3DVar
ERA-Interim	ECMWF	3	2006	80	4DVar
JRA-55	JMA	3	2009	55	4DVar

^a^The generation index (GI) and information about horizontal resolution and assimilation technique are taken from Dee et al. (2015), while the model vintage (i.e., the fixation date of the agency’s operational model) has been extracted from the reanalysis-specific reference articles and differs from Dee et al. (2015) in individual cases. Yet inaccessible models (e.g., MERRA-2) and twentieth-century reanalyses that assimilate surface observations only (e.g., Compo et al. 2011) are not tabulated. We have also omitted citations of reanalyses that are not examined in the frame of the present work

^b^ *Abbreviations* *NCEP* National Centers for Environmental Prediction, *ECMWF* European Centre for Medium-Range Weather Forecasts, *ERA* ECMWF Reanalysis, *JRA-25/JRA-55* Japanese 25-year/55-year Reanalysis, *JMA* Japan Meteorological Agency, *MERRA* Modern-Era Retrospective Analysis for Research and Applications, *GMAO* Global Modeling and Assimilation Office, *CFSR* NCEP Climate Forecast System Reanalysis

^c^ *Abbreviations* *3DVar/4DVar* 3D/4D Variational Assimilation, *IAU* Incremental Analysis Update

Previous assessments of atmosphere-driven nutations have relied heavily on NCEP’s first-generation reanalysis R1, whose physical formulation and relatively coarse resolution (2°–2.5° for surface and vertical parameters) date back to 1995. Bizouard et al. (1998), Yseboodt et al. (2002), and Brzeziński et al. (2004) derived R1-related estimates at tidal frequencies for particular reanalysis periods, while Koot and de Viron (2011) additionally analyzed NCEP R2 and the second-generation ERA-40 model over a common time span from 1979 to 2002. As a sole modern-day reanalysis, ERA-Interim (henceforth ERA) has been subject to an evaluation of nutation signals by Brzeziński (2011). Ignoring differences due to varying analysis periods, the S$_1$ estimates of these studies exhibit a fair agreement, roughly at 20–30 $\upmu$as, though individual outliers exist. In an attempt to document the same level of agreement or even further convergence for third-generation reanalyses, the present work derives nutation values for ERA and its contemporaries MERRA (Rienecker et al. 2011) and CFSR (Saha et al. 2010).

Operational models, designed for weather prediction on a daily basis, may be thought to be less suitable for long-term nutation studies. Indeed, S$_1$ estimates of Yseboodt et al. (2002) from early operational systems of ECMWF, NCEP, and JMA differ by as much as 70 $\upmu$as, suggesting that the regular changes to the model and assimilation technique within each agency are capable of introducing artificial tidal variability (Koot and de Viron 2011). To some extent, though, the results of Yseboodt et al. (2002) are affected by time series limitations, e.g., data gaps of up to 30 % or occasionally short record lengths (3 years). If provided continuously, operational datasets might, in fact, still be proper vehicles for tidal studies, as the underlying analysis systems are optimized to represent the atmospheric variability on short timescales and as they are also readily adaptable to the introduction of new data types. By contrast, reanalyses assimilate an evolving observation record through a predefined framework and are thus prone to spurious variabilities (Dee et al. 2015). Moreover, and in the context of nutation, most model updates involve resolution and orography changes that possibly cause local discontinuities in continental surface pressure but are of no immediate consequence for all other components of the global S$_1$ tide (i.e., the wind signal at higher altitudes, the pressure tide over the ocean, and, thus, the full oceanic S$_1$ variability).

**Table 2:** Grid specifications of the utilized atmospheric assimilation models

Data stream	Time span	$\Delta {t}$ (h)	Horizontal resolution	
			Surface	Pressure level
MERRA, assimilated state	1994–2013	3	$1.25^{\circ }$	$1.25^{\circ }$
CFSR, analysis & 3-h forecast	1994–2010	3	$0.5^{\circ }$	$2.5^{\circ }$
ERA-Interim, analysis	1994–2013	6	$0.5^{\circ }$	$2.0^{\circ }$
ECMWF, operational analysis	2004–2013	6	$1.0^{\circ }$	$1.0^{\circ }$

In all four cases, the vertical data are discretized on 25 isobaric levels

Table 2 summarizes the main specifications of the globally gridded datasets from MERRA, CFSR, ERA, and the ECMWF operational model, denoted as EC-OP in the following. The analysis was initially conceived for the period of 2004–2013 (Schindelegger et al. 2015) but extended in retrospect to a 20-year window (1994–2013) for the three reanalyses. CFSR constitutes an exception, having been produced as a genuine, constant-model reanalysis until the end of 2010 with subsequent operational extensions that were, however, disregarded in the frame of the present work. We extracted standard 6-h analysis fields from the respective data archives for both ECMWF models as well as CFSR, whereas 3-h analysis/forecast combinations, designated as *assimilated states*, were utilized for MERRA. This high-resolution dataset has been a source of continued Earth rotation research at TU Wien, comprising also investigations of the semidiurnal atmospheric tide S$_2$, which is not properly resolved by four-times-daily analysis products. To prepare for further subsequent work on S$_2$, we also interlaced 3-h forecast data to the CFSR analysis fields after verifying that their inclusion had no evident effect on the S$_1$ signature in surface pressure and nutation estimates.

Atmospheric excitation is classically inferred from the two components of AAM (atmospheric angular momentum), comprising effects both due to particle movement (wind or motion term) and redistribution of matter (pressure or mass term). The evaluation of the wind term involves vertical integration over an appropriate number (>15) of isobaric levels and is thus computationally intensive. Yet, the higher-altitude horizontal convection associated with S$_1$ constitutes a robust, large-scale signal that is integrated with sufficient accuracy from comparatively coarse mesh sizes of about 2°; cf. Table 2. Given the pronounced small-scale (and possibly subgrid-scale) characteristics of the diurnal surface pressure variability over landmasses (e.g., Li et al. 2009), the mass component of AAM is preferably deduced from better resolved surface grids, although limitations are imposed by the intrinsic model resolution and our hardware resources. MERRA’s assimilated states of surface pressure are distributed at 1.25° in latitude and longitude, whereas 0.5° grids could be utilized for CFSR and ERA. Note that the native 80-km spacing of ERA (Table 1) is somewhat coarser than 0.5°, suggesting that these data were interpolated during the assignment process. By contrast, the chosen 1° mesh for the operational data is a largely downsampled version of the model’s fine intrinsic discretization, having improved in resolution from 40 km in 2004 to 16 km at the end of 2013.

**Fig. 2:**
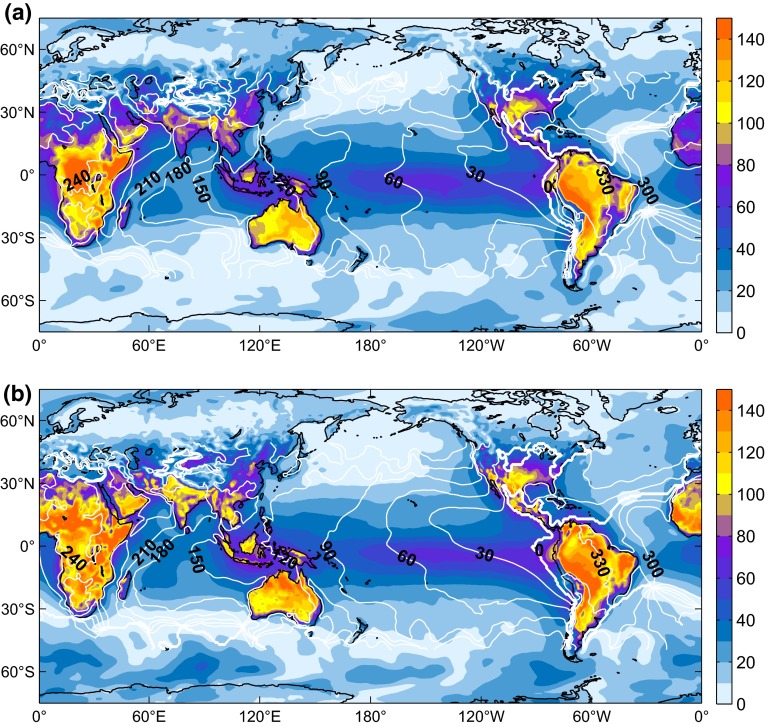
Cotidal charts of S$_1(p)$, the principal diurnal tide in surface pressure: **a** 2004–2013 average obtained from 3-h MERRA assimilation data; **b** 2001–2010 average from 3-h CFSR analysis/forecast combinations. *Color-filled* contours show amplitudes in Pa and *white isolines* indicate phase lags relative to Greenwich noon every 30$^\circ$, a lag of 0$^\circ$ being shown by the *bold line*

A preliminary comparison of two reanalyses with regard to their diurnal cycle is shown in Fig. 2

in the form of cotidal S$_1(p)$ charts for MERRA and CFSR. Mean amplitude and phase lag values were obtained from a standard least-squares tidal analysis of 10-year pressure time series at each grid point location. These climatologies echo the well-known spatial characteristics of the diurnal barometric tide (see Ray and Ponte 2003, and references therein) but also exemplify that its representation in global analysis models can diverge. CFSR suggests higher S$_1$ amplitudes almost throughout the world, particularly over landmasses in latitudes lower than 30$^\circ$ and in valleys for which the local diurnal oscillation is not resolved by MERRA (e.g., Sierra Nevada). Large regional-scale differences over flatter terrain (e.g., Sahara, Central Africa, India) portend to difficulties in the models to represent the significant diurnal boundary-layer effects driven by sensible and latent heating from the ground (Dai and Wang 1999). These non-migrating components can be excluded from the tidal spectrum by performing a Fourier decomposition of S$_1(p)$ by wavenumber *s* (Chapman and Lindzen 1970) and retaining only the main Sun-synchronous, migrating S$_1^1$ tide ($s=1$). Forced by tropospheric absorption processes, S$_1^1$ corresponds to a longitudinally uniform wave that is clearly evident in Fig. 2 over oceanic areas. Latitudinal profiles of this migrating tide from MERRA and CFSR exhibit a fair agreement (not shown), although equatorial peak amplitudes differ slightly (64.1 Pa for MERRA, 67.2 Pa for CFSR) and an overestimation of about 10 Pa at latitudes close to 60$^\circ$ S can be observed for the CFSR solution. These discrepancies in pressure are likely to have a bearing on the simulation of the oceanic S$_1$ tide.

## Atmospheric Contributions to Nutation

### Implementation

Computation of the vector equatorial AAM mass term $\tilde{H}^p=H_x^p+iH_y^p$ (complex notation) involves weighted-area double integrals of surface pressure, whereas for the motion term $\tilde{H}^w=H_x^w+iH_y^w$ a full 3D summation of geometrically weighted horizontal winds is required. We applied the respective standard formulas, given, e.g., in Sect. 2.5 of Schindelegger et al. (2013) on the gridded datasets of Table 2, with lower boundaries in the vertical integration taken from the model-specific topographies. A priori corrections to the mass terms for an isostatic (inverted barometer, IB) ocean response to air pressure variations were categorically avoided, as the oceanic S$_1$ tide is a dynamic phenomenon that will be rigorously estimated in Sect. 4.

**Table 3:** Numerical values for use in the resonance formula (Eq. 2) following Koot and de Viron (2011)^a^

Mode	${\tilde{\sigma}}$ (cpsd)		${\tilde{N}}^p \times 10^3$		${\tilde{N}}^w \times 10^3$	
	Real	Imag	Real	Imag	Real	Imag
CW	0.00251794	−0.00000564	2.561471	−0.004259	3.702480	$-$0.000004
FCN	−1.00232436	0.00002539	0.235658	0.000612	0.000973	$-$0.000009

^a^Theoretical, complex-valued frequencies of CW and FCN are given in the Earth-fixed frame and correspond to a terrestrial period of 396.06 solar days with quality factor $Q=223$ for the CW and a celestial period of $-429.05$ solar days with a (terrestrial) quality factor of $Q=19736$ for the FCN resonance

$\tilde{H}^{p,w}$ are the basic excitation quantities that can be related to nutation through a proper dynamical theory. Sasao and Wahr (1981) devised corresponding expressions for the geophysically driven nutation from the angular momentum balance equations of a coupled two-layer Earth, comprising mantle and a fluid core which are allowed to deform elastically under the action of body tides and atmospheric (oceanic) loads at the Earth’s surface. Brzeziński (1994) reformulated this pilot equation to a practicable broad-band excitation scheme for both nutation and polar motion. Yet, for reasons of consistency, the comparison of geophysical model estimates with the S$_1$ post-fit correction terms of MHB and Koot et al. (2010) is better accomplished through the excitation scheme of Koot and de Viron (2011). Their formalism conforms to MHB’s nutation theory for an up-to-date Earth model with inner core dynamics and anelastic properties. Perturbations $\tilde{n}\left( \sigma \right) =\delta X+i\delta Y$ of the celestial pole offsets in *X* and *Y* in response to changes of AAM at some Earth-referred, retrograde diurnal frequency $\sigma$ (in cycles per sidereal day, cpsd) are modeled as

1 $$\begin{aligned} \tilde{n}\left( \sigma \right) = -\tilde{T}^p\left( \sigma \right) \frac{\tilde{H}'^{p}\left( \sigma \right) }{\Omega \left( C-A\right) }-\tilde{T}^w\left( \sigma \right) \frac{\tilde{H}'^{w}\left( \sigma \right) }{\Omega \left( C-A\right) } \end{aligned}$$

where $\varOmega$ is the nominal sidereal angular velocity, *A* and *C* denote the equatorial and polar principal moments of inertia of an axisymmetric solid Earth (i.e., mantle and crust), and $\tilde{T}^{p,w}\left( \sigma \right)$ are transfer functions describing Earth’s nutation response to atmospheric forcing as conveyed by the periodic terms $\tilde{H}'^{p,w}\left( \sigma \right)$, which are defined below. The transfer functions read

2 $$\begin{aligned} \tilde{T}^{p,w}\left( \sigma \right) = \sum _{i=1}^{4} \frac{{\tilde{N}}_i^{p,w}}{\sigma -{\tilde{\sigma}} _i} \end{aligned}$$

comprising resonances at the frequencies ${\tilde{\sigma}} _i$ (cpsd) of the four rotational normal modes of a three-layer Earth: the Chandler wobble (CW), the free core nutation (FCN), the free inner core nutation, and the inner core wobble (Koot and de Viron 2011). The strengths of these resonances upon mass and motion excitation are characterized by the coefficients ${\tilde{N}}_i^{p,w}$, specified in Table 3 with a truncation at $i=2$ that retains CW and FCN and excludes the inner core modes without loss of accuracy. If viewed from the surface of the rotating Earth, the FCN occurs as retrograde nearly diurnal oscillation, thus providing significant enhancement to excitation effects associated with atmosphere–ocean dynamics at S$_1$ and adjacent tidal lines. Note, however, that nutations are much more efficiently driven by the mass term than by relative particle motion (Sasao and Wahr 1981; Brzeziński 1994); cf. also the excess of ${\tilde{N}}^p$ at the FCN frequency relative to ${\tilde{N}}^w$ by a factor of 200 (Table 3).

The forcing terms $\tilde{H}'^{p,w}\left( \sigma \right)$ in Eq. (1) are complex coefficients of time dependence $\propto \mathrm {e}^{i\sigma t}$ as seen from the rotating reference frame (Koot and de Viron 2011). Yet, the amplitudes of these sinusoids are typically estimated in inertial space after translating the terrestrial AAM time series $\tilde{H}\left( t\right)$ to their celestial counterparts $\tilde{H}'\left( t\right)$ via the demodulation (Brzeziński 1994)

3 $$\begin{aligned} \tilde{H}'\left( t\right) = -\tilde{H}\left( t\right) \mathrm {e}^{i \left[ \Omega \left( t-t_0\right) +\Phi _0 \right] } \end{aligned}$$

that is applicable to both pressure and wind effects (respective superscripts have been omitted for brevity). The exponent represents a sufficiently accurate linear approximation for the Greenwich sidereal time (Bizouard et al. 1998), employing a phase offset of $\varPhi _0$ referred to $t_0$ at 12 h UT1, 1 January 2000 (J2000.0). The conventional expression for the Earth rotation angle of the IERS (Petit and Luzum 2010) is compatible with Eq. (3) and implies $\varPhi _0 = 0.7790572732640$ rad as well as $\varOmega = \left( 2\pi r\right)$ rad per solar day, where $r = 1.00273781191135448$ scales solar to sidereal time intervals; see also Koot and de Viron (2011).

The demodulation procedure preserves amplitudes but maps the retrograde ($\sigma < 0$) quasi-diurnal spectral components to low frequencies, with the center frequency $\varOmega$ (i.e., the K$_1$ band) shifted to zero and S$_1$ appearing as prograde annual line in the celestial frame. Dominant (intra-)seasonal signals in the original AAM series are mapped to high frequencies in space and are efficiently removed through filtering (Bizouard et al. 1998). To this end, we applied an idealized rectangle filter with cutoff at 20 cpy (cycles per year) on the frequency transform of $\tilde{H}'\left( t\right)$ and resampled the proper inverse transform in the time domain at daily intervals. Experiments with more customary time domain filters and a range of reasonable cutoff frequencies testified to the insensitivity of our nutation results to details in the filtering strategy.

**Table 4:** Multipliers of the fundamental arguments of nutation terms that apply to atmospheric tidal lines^a^

Term	Fundamental arguments					Period	Phase
	*l*	$l'$	*F*	*D*	$\Omega$	(solar days)	(°)
S$_1$	0	1	0	0	0	365.260	357.529
$\psi _1$	0	−1	0	0	0	−365.260	−357.529
P$_1$	0	0	2	−2	2	182.621	−159.067
$\phi _1$	0	0	−2	2	−2	−182.621	159.067
$\pi _1$	0	1	2	−2	2	121.749	198.462

^a^Periods and phases (referred to J2000.0) result from the linear combinations of series expansions as given by Petit and Luzum (2010) for each argument. The table is identical to Table 1 of Koot and de Viron (2011) except for the $\phi _1$ term

To convert the low-frequency, quasi-harmonic celestial AAM time variability associated with S$_1$ and its seasonal modulations to periodic circular components $\tilde{H}'\left( \sigma \right)$ for use in Eq. (1) we imposed a Fourier decomposition

4 $$\begin{aligned} \tilde{H}'\left( t\right) = -i\sum _{j=1}^{5} \tilde{a}_j \mathrm {e}^{i\left[ \nu _j \Omega \left( t-t_0\right) +\varphi _j\right] } + \tilde{c} \end{aligned}$$

on the complex-valued filter output. The non-dimensional frequencies $\{\nu _j\}_{j=1}^{5}$ of the demodulated tidal constituents $\{\mathrm {S}_1,\psi _1,\mathrm {P}_1,\phi _1,\pi _1\}$ are $\{1,-1,2,-2,3\}/366.26$ (Koot and de Viron 2011), and respective phase values $\{\varphi _j\}_{j=1}^{5}$ agree with those from the corresponding lunisolar nutation terms. The $\varphi _j$ are readily computed from the fundamental arguments of nutation theory (Petit and Luzum 2010), using the integer multipliers specified in Table 4. A standard least-squares fit of $\tilde{H}'\left( t\right)$ onto these basis functions provides the unknown parameters $\tilde{a}_j$ composed of real (in-phase, ip) and imaginary (out-of-phase, op) parts that form the complex-valued forcing terms in Eq. (1) at discrete terrestrial frequencies $\sigma _j = \nu _j-1$ (cpsd). The constant pole offset contribution from the K$_1$ tide, conveyed by the estimated $\tilde{c}$ term, was excluded from further consideration, as were the minute secular (precession) contributions associated with the time derivatives of nutation arguments.

The harmonic decomposition in Eq. (4) exactly follows the model of Koot and de Viron (2011) except for our inclusion of the $\phi _1$ component at $-2$ cpy that suggests some inter-annual modulation of the thermal S$_1$ tide. While the existence of such a modulation is debatable (Bizouard et al. 1998), its impact on nutation is at the level of 10 $\upmu$as and thus comparable to the usually modeled $\pi _1$ term. Moreover, for numerical reasons, we performed the least-squares fit on the basis of prescaled AAM time series $\tilde{H}'\left( t\right) /\left( \Omega \left( C-A\right) \right)$ (Eq. 1) in units of $\upmu$as, similar to the classical “celestial effective angular momentum functions” of Brzeziński (1994). The excitation scheme of this author was tested briefly and found to yield nutation results well within 5 % of the estimates from the above formalism.

### Results

**Fig. 3:**
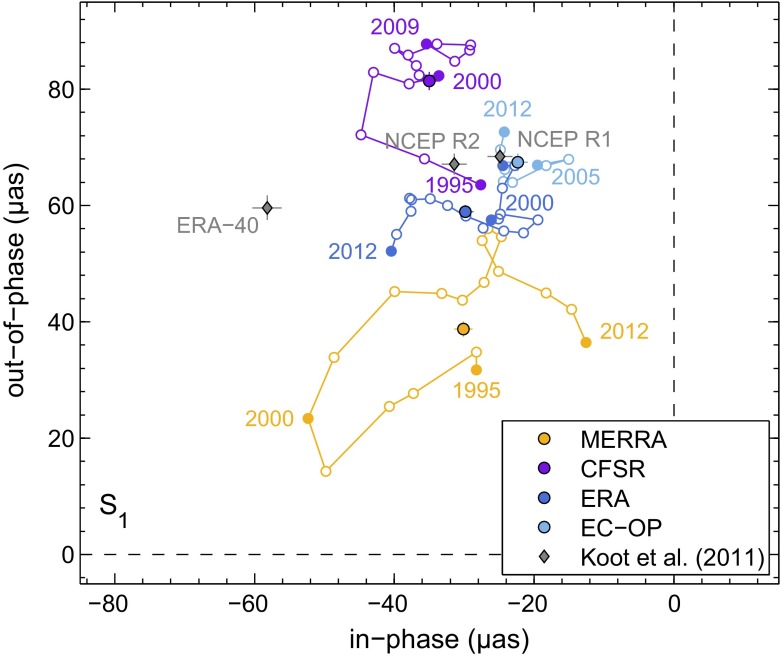
Total (pressure plus wind) atmospheric contribution to the prograde annual nutation computed for MERRA (*orange*, 1994–2013), CFSR (*purple*, 1994–2010), ERA (*dark blue*, 1994–2013), and EC-OP (*light blue*, 2004–2013), using the fundamental arguments and sign conventions of Sect. 3.1. Mean estimates with 3$\sigma$ formal errors are displayed as *single* markers and compared to the S$_1$ results from Koot and de Viron (2011) from earlier generation reanalyses for the period 1979.0–2002.7 (*gray* markers). The temporal variability of our estimates is disclosed by 3-year sliding window values for each atmospheric model (*colored curves*)

Figure 3 displays the superimposed pressure and wind nutation estimates

$\tilde{n}\left( \sigma \right)$ in the prograde annual band, both as mean contributions over the model-specific time spans as well as yearly values obtained from repetitions of the analysis in Sect. 3.1 with a 3-year sliding window. Consistent with illustrations in Yseboodt et al. (2002) and Koot and de Viron (2011), little agreement is seen between all S$_1$ curves in terms of their inter-annual variability, even though the post-2004 estimates are stable within 20 $\upmu$as for each model. Roughly 75 % of the observed fluctuations are driven by the pressure term and likely relate to random perturbations of the second-order tesseral harmonic in surface pressure, i.e., the only component of the S$_1\left( p\right)$ wave that efficiently excites Earth’s nutational motion. Amplitudes of this mode do not exceed 10 Pa, so its representation in atmospheric assimilation systems is prone to noise interferences.

By contrast, a signal with a possibly physical origin is evident for MERRA during 1997–2001, coinciding in time with a peak El Niño event in 1997/1998 and subsequent cold La Niña conditions up to 2001; cf., e.g., the Oceanic Niño Index tabulated at http://www.cpc.noaa.gov/products/analysis_monitoring/ensostuff/ensoyears.shtml (accessed 29 September 2015). The warm phase of ENSO (El Niño–Southern Oscillation) has been previously suggested to alter the radiative forcing of the solar tide in the troposphere (Lieberman et al. 2007), thereby providing significant enhancement to diurnal pressure oscillations across the Pacific (Vial et al. 1994). The response of the climate system to ENSO events is, however, not restricted to the Tropics but can entail atmospheric circulation changes in higher latitudes that may ultimately couple to nutation; see similar conjectures in Yseboodt et al. (2002). Assessing whether the irregular nutation changes from MERRA in Fig. 3 are linked to ENSO or merely represent spurious variabilities in the wake of observing system changes (Robertson et al. 2011) is beyond the scope of this study, though. We will in fact avoid these signals in our selection of the mean analysis window below.

In terms of multi-year nutation averages, the ECMWF-based solutions agree particularly well with each other and with Koot and de Viron (2011)’s results for the first-generation NCEP models from 1979 to July 2002. The ERA-40 estimate of these authors is anomalous in the ip component ($-58.2$ $\upmu$as) due to erratic S$_1$ variations up to the mid-1990s (cf. Fig. 2 of Koot and de Viron 2011). Disregarding the impact of the “ENSO swerve” on the MERRA solution, the only nutation anomaly in the present work is a large CFSR estimate, with op pressure term values (50 μas as from 1998) exceeding the predictions from other reanalyses by 20–30 $\upmu$as. This overestimation traces back to the dubious CFSR pressure oscillations of more than 40 Pa in the Southern Ocean (Fig. 2), a region that is void of conventional in situ observations and sensitive to the details of radiance data assimilation. Note also that the quality with which atmospheric tides can be represented in analysis systems is tied to the time step of radiative processes (Poli et al. 2013). Trading off computational costs and a fine spatial resolution, CFSR integrates its longwave radiation parameterization every 3 h (Saha et al. 2010), significantly coarser than the hourly time step recommended by Poli et al. (2013) and employed within MERRA, ERA, and EC-OP.

**Table 5:** Periodic atmospheric contributions to nutation ($\upmu$as, with 1$\sigma$ errors) computed from the model-specific pressure and wind terms over an analysis period of 2004–2013^a^

Term	Model	Pressure		Wind		Total	
		ip	op	ip	op	ip	op
S$_1$ ($+1$ year)	MERRA	$-25.9\pm 0.4$	$18.1\pm 0.4$	$4.0\pm 0.2$	$27.6\pm 0.2$	$-21.9\pm 0.5$	$45.8\pm 0.5$
	MERRA^b^	$-30.8\pm 0.5$	$23.0\pm 0.5$	$5.0\pm 0.2$	$26.6\pm 0.2$	$-25.8\pm 0.6$	$49.7\pm 0.6$
	CFSR^b^	$-32.3\pm 0.6$	$57.5\pm 0.6$	$-2.0\pm 0.2$	$30.0\pm 0.2$	$-34.3\pm 0.6$	$87.6\pm 0.6$
	ERA	$-31.8\pm 0.5$	$31.7\pm 0.5$	$-4.4\pm 0.2$	$25.8\pm 0.2$	$-36.1\pm 0.5$	$57.5\pm 0.5$
	EC-OP	$-21.9\pm 0.5$	$43.8\pm 0.5$	$-0.5\pm 0.2$	$23.6\pm 0.2$	$-22.4\pm 0.5$	$67.5\pm 0.5$
$\psi _1$ ($-1$ year)	MERRA	$-30.1\pm 5.9$	$-44.5\pm 5.9$	$-2.5\pm 0.3$	$-3.0\pm 0.3$	$-32.6\pm 5.9$	$-47.5\pm 5.9$
	MERRA^b^	$-45.1\pm 6.5$	$-48.4\pm 6.5$	$-2.7\pm 0.4$	$-3.6\pm 0.4$	$-47.9\pm 6.5$	$-52.0\pm 6.5$
	CFSR^b^	$-58.2\pm 7.3$	$0.4\pm 7.3$	$0.6\pm 0.3$	$-3.2\pm 0.3$	$-57.5\pm 7.3$	$-2.9\pm 7.3$
	ERA	$-79.3\pm 6.1$	$22.0\pm 6.1$	$-4.0\pm 0.3$	$-0.7\pm 0.3$	$-83.3\pm 6.1$	$21.3\pm 6.1$
	EC-OP	$-50.8\pm 6.2$	$23.6\pm 6.2$	$-3.1\pm 0.3$	$0.9\pm 0.3$	$-54.0\pm 6.2$	$24.5\pm 6.2$
P$_1$ ($+{1}/{2}$ year)	MERRA	$-10.1\pm 0.3$	$0.1\pm 0.3$	$7.1\pm 0.2$	$42.1\pm 0.2$	$-3.0\pm 0.3$	$42.2\pm 0.3$
	MERRA^b^	$-10.3\pm 0.3$	$1.2\pm 0.3$	$7.1\pm 0.3$	$41.1\pm 0.3$	$-3.2\pm 0.4$	$42.3\pm 0.4$
	CFSR^b^	$-6.9\pm 0.3$	$7.0\pm 0.3$	$5.1\pm 0.2$	$35.6\pm 0.2$	$-1.9\pm 0.4$	$42.6\pm 0.4$
	ERA	$-17.3\pm 0.3$	$0.9\pm 0.3$	$13.5\pm 0.2$	$45.8\pm 0.2$	$-3.8\pm 0.3$	$46.7\pm 0.3$
	EC-OP	$-17.0\pm 0.3$	$2.5\pm 0.3$	$11.2\pm 0.2$	$41.5\pm 0.2$	$-5.8\pm 0.3$	$44.1\pm 0.3$
$\phi _1$ ($-{1}/{2}$ year)	MERRA	$-2.1\pm 0.8$	$-6.1\pm 0.8$	$0.9\pm 0.2$	$-3.0\pm 0.2$	$-1.2\pm 0.8$	$-9.1\pm 0.8$
	MERRA^b^	$-1.8\pm 0.9$	$-5.9\pm 0.9$	$0.7\pm 0.3$	$-3.3\pm 0.3$	$-1.1\pm 0.9$	$-9.3\pm 0.9$
	CFSR^b^	$-17.3\pm 1.0$	$1.9\pm 1.0$	$-2.1\pm 0.2$	$-1.4\pm 0.2$	$-19.3\pm 1.0$	$0.6\pm 1.0$
	ERA	$-12.2\pm 0.8$	$-8.9\pm 0.8$	$-2.5\pm 0.2$	$-4.9\pm 0.2$	$-14.7\pm 0.8$	$-13.9\pm 0.8$
	EC-OP	$1.8\pm 0.8$	$-13.0\pm 0.8$	$0.7\pm 0.2$	$-5.7\pm 0.2$	$2.5\pm 0.9$	$-18.7\pm 0.9$
$\pi _1$ ($+{1}/{3}$ year)	MERRA	$-2.6\pm 0.2$	$4.1\pm 0.2$	$1.9\pm 0.2$	$-1.4\pm 0.2$	$-0.7\pm 0.3$	$2.6\pm 0.3$
	MERRA^b^	$-2.3\pm 0.2$	$4.0\pm 0.2$	$2.3\pm 0.3$	$-1.6\pm 0.3$	$0.0\pm 0.3$	$2.4\pm 0.3$
	CFSR^b^	$-2.1\pm 0.2$	$3.3\pm 0.2$	$2.1\pm 0.2$	$-0.7\pm 0.2$	$-0.1\pm 0.3$	$2.6\pm 0.3$
	ERA	$-2.5\pm 0.2$	$2.8\pm 0.2$	$1.1\pm 0.2$	$0.3\pm 0.2$	$-1.4\pm 0.3$	$3.0\pm 0.3$
	EC-OP	$-2.2\pm 0.2$	$2.7\pm 0.2$	$1.1\pm 0.2$	$-0.1\pm 0.2$	$-1.1\pm 0.3$	$2.7\pm 0.3$

^a^In- and out-of-phase components are referred to the fundamental arguments of nutation (Table 4) and the sign convention is that of Koot and de Viron (2011)

^b^Average values over the period 2004–2010

Numerical results for our nutation analysis are reported in Table 5, using—with some exceptions—an averaging period from 2004 to 2013 that has been specified after consulting station tide determinations in next section. If the somewhat deficient CFSR results are discarded, S$_1$ estimates from third-generation reanalyses and EC-OP deviate from each other by less than 22 $\upmu$as, which slightly betters the agreement noted by Koot and de Viron (2011) and conforms with the threefold VLBI SD in the prograde annual band (Fig. 1). We have also assessed the stability of the S$_1$ peak in terms of its frequency through a Morlet wavelet analysis of demodulated filter residuals $\tilde{H}'$. Deviations from the nominal S$_1$ ridge at 365.26 days (solar days) are well within 5 days for all datasets except for MERRA, which exhibits a transition from 355 days in 1998 to 385 days in 2002 before leveling off exactly at the annual period (not shown). These minor fluctuations contrast with the 30 day range deduced by Dehant et al. (2003) on the basis of NCEP R1 data during 1958–1999. We surmise, however, that the estimate of Dehant et al. (2003) is less reliable due to the inclusion of reanalysis products prior to 1979, i.e., a period that lacks both satellite retrievals and a broad network of in situ pressure observations in the southern hemisphere.

Our nutation results for the minor solar constituents ($\psi _1$, P$_1$, $\phi _1$, $\pi _1$) can be compared with estimates tabulated in Bizouard et al. (1998), Yseboodt et al. (2002), Brzeziński et al. (2004), or Koot and de Viron (2011). Here, we only point out that the harmonics fitted to the four atmospheric datasets agree well for P$_1$ and $\pi _1$ but differ substantially in the $\psi _1$ band, which is of interest for studies of the FCN and Earth’s internal properties (Dehant and Defraigne 1997; Koot and de Viron 2011). Temporal variations of this tide can be large ($>100$ $\upmu$as for individual models), and periods of its wavelet ridge vary within 30 days, probably as a reflection of a strong stochastic atmospheric influence. Nonetheless, a comparatively good inter-model agreement is found for the ip component of $\psi _1$ ($\sim$50 $\upmu$as).

### Validation of Pressure Tides against In Situ Data

**Fig. 4:**
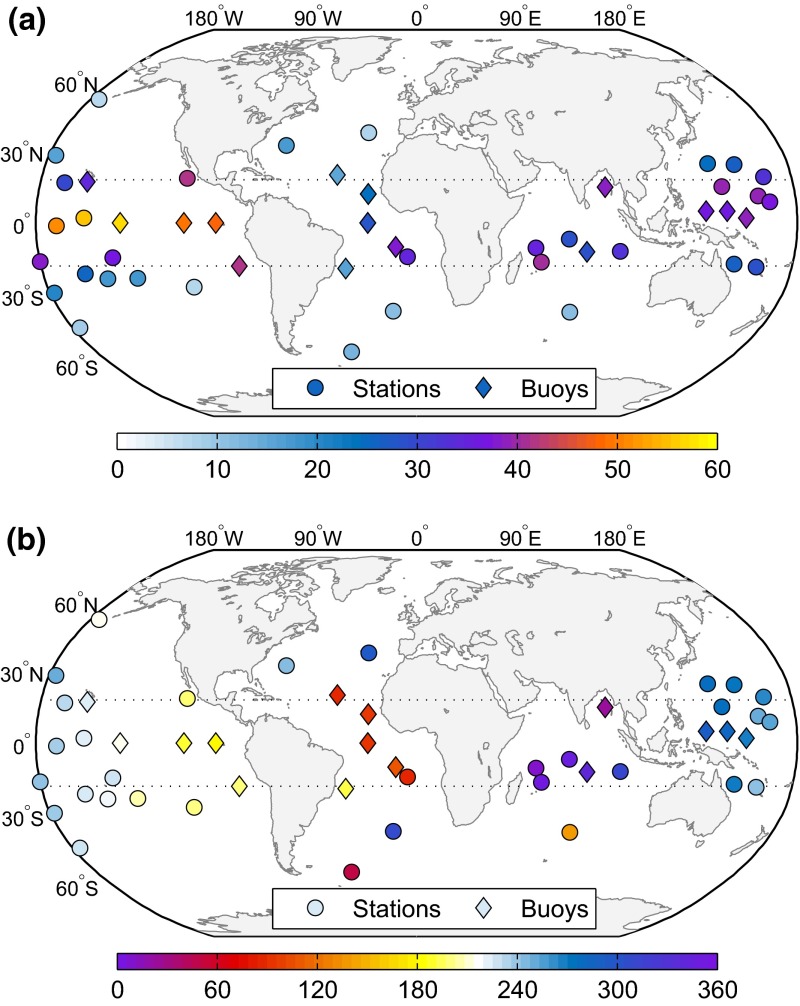
**a** Amplitudes (Pa) and **b** Greenwich phase lags (°) of the S$_1(p)$ tide, as deduced for 50 ground truth stations, with buoy locations shown as diamond markers. *Dotted lines* indicate latitudes of 18$^\circ$ used as cutoff in Fig. 5

Figure 4 displays the tidal constants of 50 island and buoy barometers which were assembled within the frame of this study for validation purposes. Information about the AAM-related degree 2 harmonic is inaccessible by means of such scattered data, yet the pressure tide forcing of the ocean model can be tested locally;

cf. Ray and Ponte (2003). We have placed this analysis in the atmospheric section of the paper to underpin our choice of the averaging period in Table 5.

The high-quality backbone of our compilation comprises 16 S$_1$ estimates from Ray (1998a). We excluded nine solar determinations of this author (e.g., three Hawaiian sites, Ascension, or Tahiti) as they were inconsistent with nearby open-ocean estimates from smaller islands and buoys, presumably as a result of the latent and sensible heat flux over larger landmasses. 18 additional S$_1$ determinations come from Schindelegger and Dobslaw (2016), who tidally analyzed hourly and 3-h synoptic sea level pressure observations from the ISD (Integrated Surface Database, Smith et al. 2011) to extract the much smaller lunar semidiurnal L$_2$ tide. Again, care was exercised in selecting stations at sufficiently small islands and atolls. The final subset of estimates, also derived by Schindelegger and Dobslaw (2016), comprises 16 buoy locations that are part of the Tropical Moored Buoy System (McPhaden et al. 2010). Further densifications were attempted but led to clusters and subsequent biases in the statistics given below. The median time series length is 6 years, the maximum is 26 years (Bermuda), and short time spans of only 2 years occurred for eight sites, most of them being buoys.

**Fig. 5:**
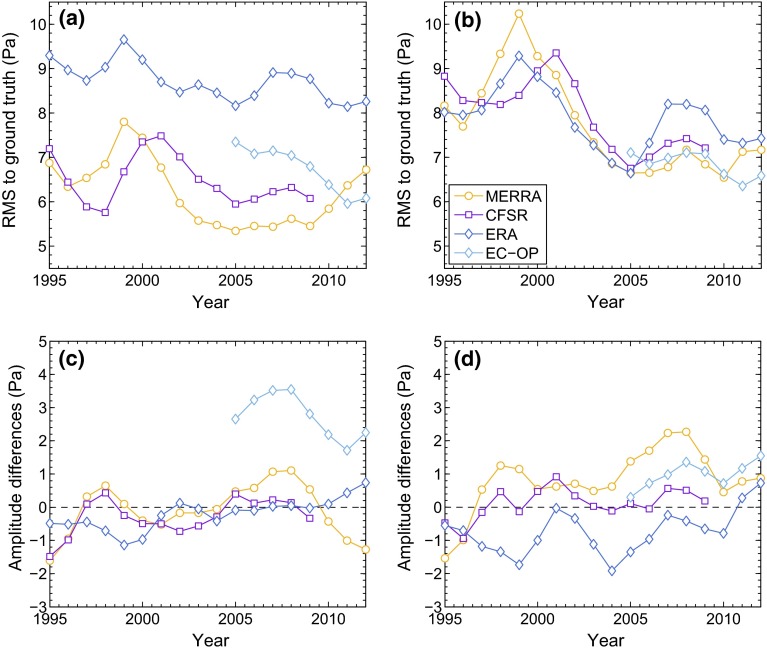
RMS statistics (*upper panels* **a**, **b**) and amplitude differences (*lower panels* **c**, **d**) of model surface pressure tides against two versions of our S$_1$ ground truth compilation: **a**, **c** full network as shown in Fig. 4; **b**, **d** non-equatorial subset of 20 station with sites at latitudes below 18$^{\circ }$ excluded. The time dimension in the plots is due to the model S$_1\left( p\right)$ solutions having been computed as 3-year averages in yearly steps, while the ground truth network has been kept constant as a climatological mean. The displayed RMS and amplitude differences (model-minus-station) are global averages over 50 and 20 sites, respectively

Atmospheric model pressure values were evaluated by bilinear interpolation at the locations of the 50 ground truth stations and tidally analyzed in a moving 3-year window. RMS statistics and globally averaged amplitude differences from the comparison of these windowed S$_1$ solutions to the in situ estimates (non-windowed) are shown in Fig. 5,

both for the full global network as well as for a subset of 20 stations excluding latitudes lower than 18$^{\circ }$. This restriction avoids an over-emphasis on the large migrating pressure tide near the equator and tests smaller signals in mid-latitudes, i.e., regions of increased importance for nutation. The resulting network (Fig. 4) is, however, very sparse and dominated by 13 stations in the Pacific.

In Fig. 5a (full compilation), ERA features the largest RMS misfits at about 9 Pa, accumulated through a significant overestimation of S$_1\left( p\right)$ in the Tropics and an underestimation of the tidal amplitude elsewhere (Fig. 5d). These deficiencies can be expected to lead to a less reliable oceanic S$_1$ tide. For equatorial Pacific stations, the second ECMWF dataset EC-OP also produces an excess in amplitude (by about 10 Pa) that maps into Fig. 5c but has no effect on the reduced network (Fig. 5d). Yet, median and normalized RMS differences are generally better for EC-OP than for the probed reanalyses and highlight the accuracy of the operational solution in a global domain. The CFSR statistics are comparable to those of MERRA and EC-OP, evidently unaffected by the southern hemispheric pressure anomalies (Sect. 3.2) as these regions are not sampled by our ground truth network.

The 1998 El Niño and its subsequent reversal during 1999–2001/2002 introduce irregular tidal behavior and larger RMS values in all three reanalyses when validated against the climatological in situ solution. Following our prescription of minimal inter-annual S$_1$ variability, we excluded model data up to 2002 from further consideration. A sufficiently long averaging period, required to somewhat conform with the 20-year mean S$_1$ fit of MHB, might thus be realized by the overlapping 7-year window (2004–2010) common to all models. Disregarding the partially deficient CFSR analysis, we finally adopted the time span from 2004 to 2013 as the main analysis window for MERRA, ERA, and EC-OP, while CFSR results were averaged through 2004–2010, and MERRA excitation data for 2004–2010 were maintained as well, but only as a secondary option. This duality for MERRA is motivated by the deterioration of RMS values (Fig. 5a) and the moderate drifts in nutation estimates (Fig. 3a) as from the year 2010.

**Table 6:** S$_1\left( p\right)$ differences of the tested analysis models with 50 station tide determinations, expressed as RMS misfits (Pa), median absolute differences (MAD, Pa), and median phase differences $\Delta \varphi$ in the sense model-minus-station^a^

	RMS	MAD	$\Delta \varphi$
MERRA	5.2	5.1	6.1°
CFSR	5.9	5.9	6.1°
ERA	8.3	7.2	−5.2°
EC-OP	6.5	5.9	2.5°

^a^Model tides are 2004–2013 averages except for CFSR (2004–2010)

A brief numerical comparison of the resulting model pressure tide climatologies against the 50-station set of S$_1$ estimates is given in Table 6. Median absolute differences (MAD) are included as a more robust supplement to the averaged RMS misfits, underlining the reliability of all analysis models other than ERA. The general level of consistency with ground truth data (5–6 Pa) closely resembles values obtained by Ray and Ponte (2003), but note that these authors have additionally applied a small phase shift $\Delta \varphi$ to their model estimates. Median phase lag differences for MERRA and CFSR in Table 6 generally confirm Ray and Ponte (2003)’s assumption of $\Delta \varphi = 5^\circ$, and imposing the corresponding correction on the MERRA tide does indeed reduce the RMS misfit with station data to 4.9 Pa. We have, however, abstained from revising the tidal phases to avoid inconsistencies with the diurnal cycle in AAM.

## Numerical Modeling of the Oceanic S$_1$ Tide

### Ocean Model Configuration

DEBOT (Einšpigel and Martinec 2015) is a recently developed ephemeris-forced, barotropic time-stepping model conceived to study the effect of the ocean flow on Earth’s magnetic field. In the present work, we create a spin-off of the model’s hydrodynamic core for individual partial tides, with a rigorous treatment of SAL and a parameterized drag term to account for the conversion from barotropic waves to baroclinic internal tides (IT) over rough bottom topography. The one-layer shallow water momentum and mass conservation equations define the horizontal velocity vector $\mathbf {u}$ and the tidal surface displacement $\zeta$

5 $$\begin{aligned} \frac{\partial \mathbf {u}}{\partial t} + f \hat{z}\times \mathbf {u} =&- g\nabla \left( \zeta - \zeta _{EQ} - \zeta _{SAL} - \zeta _{MEM} - P/g\rho \right) \nonumber \\&- \frac{C_D\Vert \mathbf {u}\Vert \mathbf {u}}{H+\zeta } - \frac{C_{IT}\mathbf {u}}{H+\zeta } + A_H \nabla \cdot \varvec{\sigma } \end{aligned}$$

6 $$\begin{aligned} \frac{\partial \zeta }{\partial t} =&- \nabla \cdot \left[ \left( H+\zeta \right) \mathbf {u}\right] \end{aligned}$$

where *f* is the Coriolis parameter oriented along the local vertical unit vector $\hat{z}$, *g* is the nominal gravitational acceleration, $\nabla$ signifies the spherical del operator, *H* is the resting water depth, $\rho$ is the average density of seawater, $C_D=0.003$ denotes a dimensionless drag coefficient in the standard expression for quadratic bottom friction, and $C_{IT}$ is a location-dependent scalar (in units of m s$^{-1}$) to represent the drag due to tidal conversion. The forcing terms in the gradient operator of Eq. (5) comprise the gravitational equilibrium tide $\zeta _{EQ}$, a combination of self-attraction/loading and “memory” elevations $\zeta _{SAL}$ and $\zeta _{MEM}$ to realize the SAL scheme of Arbic et al. (2004), as well as the atmospheric pressure tide $P=\mathrm {S}_1\left( p\right)$. $A_H \nabla \cdot \varvec{\sigma }$ is a comparatively rigorous implementation of the horizontal turbulent eddy viscosity with a second-order tensor $\varvec{\sigma }$ related to the Reynolds stress tensor; see Einšpigel and Martinec (2015) for details. Here, we keep this term to eschew possible numerical instabilities in our medium-resolution runs (Egbert et al. 2004), with horizontal viscosity $A_H$ set to the widely cited value of $10^3$ m$^2$ s$^{-1}$. Our forward tidal solutions and OAM results are insensitive to the exact value of $A_H$, unless it is used as a tuning parameter of inordinately large magnitude ($\sim$10$^5$ m$^2$ s$^{-1}$); cf. Arbic et al. (2004).

Equations (5) and (6) were solved by finite difference time-stepping on a ${1}/{3}^\circ$ C-grid, covering the latitude range from $78^\circ$S to $78^\circ$N with rigid walls assumed at the top and the bottom of the domain. This setting does not allow for accurate tidal modeling in the Weddell and Ross Sea, or in the Arctic Ocean. Yet, we readily accept such high-latitude limitations given our interest in the equatorial component of Earth’s rotation that has a peak sensitivity at $45^\circ$ from the equator. The bottom topography was derived from the bedrock version of the fully global $1' \times 1'$ ETOPO1 database (Amante and Eakins 2009) by choosing average values over each ${1}/{3}^\circ$ model grid cell and setting depths between 10-m and the 0-m land–sea boundary to 10 m. Coastlines in the Antarctic come from a recent data-assimilative ocean model (Taguchi et al. 2014) and are similar to those of Padman et al. (2002), with the cavities under the floating ice shelves considered as part of the ocean domain; cf. also Arbic et al. (2004) or Carrère et al. (2012). Blocking ice shelf areas as dry cells would lead to a noticeable increase of the tidal variability in southern hemisphere waters, also amplifying the oceanic contribution to the prograde annual nutation by roughly 10 $\upmu$as in both ip and op components. However, in these simulations, also the RMS misfit of the gravitationally forced constituents (M$_2$ and O$_1$; see below) to altimetry-based reference solutions, (FES2012, Carrère et al. 2012) increases, consistent with similar control runs by Wilmes and Green (2014). We thus proceed on the assumption that vertically displaceable ice shelves allow for a more realistic account of the tides, including S$_1$.

Other aspects of the DEBOT configuration closely follow Ray and Egbert (2004). We prescribe equilibrium tidal forcing ($\zeta _{EQ}$) for M$_2$ and O$_1$ with amplitudes and solid Earth tide corrections taken from Table 1 of Arbic et al. (2004). The resulting larger-magnitude background variability appears to aid the fidelity with which S$_1$ can be simulated in various basins and bays, but an extension to more than one diurnal and semidiurnal gravitational constituent is expendable as it alters our S$_1$ OAM estimates by less than 3 %. Experiments with different equilibration periods (up to 90 days) showed that the spin-up time of the model could be reduced to 12 days with little effect (cf. Arbic et al. 2004), although in very shallow waters (Gulf of Thailand, Java Sea) the convergence of S$_1$ takes considerably more time than that of any gravitational tide, presumably due to the vagaries of the pressure forcing near landmasses (Fig. 2). With 12 days reserved for equilibration, we integrated the model in each of our runs for 40 days at a time step of 24 s, harmonically analyzing the last 28 days to deduce the tidal constants of S$_1$ (as well as M$_2$ and O$_1$) in terms of sea level elevation and barotropic volume transports $\varvec{u}H$.

### Effects of Self-Attraction and Loading (SAL)

Gravitational self-attraction and yielding of the solid Earth to the weight of the water column (Hendershott 1972) are feedback effects to the tidal dynamics and included in Eq. (5) as an additional equilibrium-like tide $\zeta _{SAL}$. This term can be related to the (unknown) local tidal elevation $\zeta$ through convolution with the global SAL Green’s function $\mathcal {G}$ (Ray 1998b)

7 $$\begin{aligned} \zeta _{SAL} \left( \phi ,\lambda \right) = \rho {{a}^2} \iint \zeta \left( \phi ',\lambda '\right) \mathcal {G}\left( \psi \right) sin\phi ' d\phi ' d\lambda ' \end{aligned}$$

where *a* is the Earth’s radius and $\psi$ measures the angular separation of $\left( \phi ,\lambda \right)$ from the load with spherical coordinates $\left( \phi ',\lambda '\right)$. For our ${1}/{3}^\circ$ model, values of $\mathcal {G}\left( \psi \right)$ were interpolated from the SAL kernel function tabulated in Stepanov and Hughes (2004). Explicit usage of Eq. (7) in the momentum equations is computationally unfeasible (Egbert et al. 2004), so alternative implementation schemes are required. To first order, the full convolution with $\mathcal {G}$ is approximated by a simple scalar multiplication $\zeta _{SAL} \approx \beta \zeta$ (Accad and Pekeris 1978), with $\beta$ usually taken to be in the range of about 0.08 to 0.12. This widely used approximation is inappropriate for all locations in the ocean (Ray 1998b) and accurate tidal modeling necessitates a more rigorous handling of the effect. In tide models forced by a suite of individual constituents, the unparameterized formalism of Eq. (7) can be applied in a comparatively simple manner via iteration, that is, repeated model runs where each simulation employs a better approximated SAL term to gradually achieve convergence between the tidal elevations and $\zeta _{SAL}$. We applied the iteration method of Arbic et al. (2004), initialized by the scalar SAL estimate using a nominal value of $\beta =0.12$ that is an appropriate choice for diurnal tides; cf. Parke (1982) and Fig. 11 of Einšpigel and Martinec (2015). Once this initial run is completed and harmonically analyzed, the tidal components (sine and cosine terms) of M$_2$, O$_1$, and S$_1$ are inserted into Eq. (7) in an intermediate offline computation to derive a first solution of $\zeta _{SAL}$ for each tidal constituent. The following simulation then time steps the sum of all partial SAL tides as well as an additional memory term (Arbic et al. 2004)

8 $$\begin{aligned} \zeta _{MEM} = \beta \left( \zeta -\zeta _{PREV}\right) \end{aligned}$$

that measures the departure of the tidal height $\zeta$ in the current (second) run from the cumulative M$_2$-O$_1$-S$_1$ elevation $\zeta _{PREV}$ in the previous (first) run. Subsequent iterations are performed in the same manner, drawing on continuously updated maps of $\zeta _{SAL}$ and $\zeta _{MEM}$.

**Table 7:** Convergence of the iterative SAL scheme in terms of global angular momentum mass integrals of the S$_1$ ocean tide^a^

	Scalar	1$\mathrm {st}$ iteration	2$\mathrm {nd}$ iteration	3$\mathrm {rd}$ iteration	Ray/Egbert
*x*	1.83 (164$^\circ$)	1.88 (168$^\circ$)	1.86 (167$^\circ$)	1.87 (167$^\circ$)	0.82 (158$^\circ$)
*y*	2.90 (287$^\circ$)	3.10 (283$^\circ$)	3.10 (283$^\circ$)	3.10 (283$^\circ$)	2.90 (306$^\circ$)
*z*	1.72 (188$^\circ$)	2.00 (181$^\circ$)	2.03 (183$^\circ$)	2.05 (183$^\circ$)	2.42 (219$^\circ$)

^a^Tidal solutions have been computed from our hydrodynamic model using atmospheric pressure forcing from Ray and Egbert (2004). Amplitudes are in units of $10^{23}$ kg m$^2$ s$^{-1}$ and cotidal phases are given relative to Greenwich noon, consistent with the Doodson convention for the S$_1$ phase as given in Ray and Egbert (2004). For the respective OAM formulas, refer to Chao et al. (1996)

The correction term in Eq. (8) guarantees rapid convergence of the SAL scheme, as exemplified by diminishing RMS discrepancies of the gravitational constituents against FES2012 tides in successive simulations. Specifically, with the choice of $\beta$ optimized for the diurnal band, our forward solutions of O$_1$ remain effectively unchanged after the first iteration, whereas sufficient accuracy for semidiurnal tides is reached after three iterations. More to the point, rapid equilibration of tidal dynamics is also observed for the radiational S$_1$ tide. Table 7 presents successively updated OAM mass values of S$_1$ as obtained from a three-times iterative DEBOT run with the pressure forcing S$_1\left( p\right)$ taken from Ray and Egbert (2004) and IT drag (cf. next section) switched off. For equatorial components in particular, the scalar approximation appears to provide reasonably accurate initial OAM estimates, deviating by no more than 5$^\circ$ in phase and less than 10 % in amplitude from the (arguably) self-consistent third iteration. Yet, the scalar SAL relation is inadequate for both the axial OAM component and the comparison of simulated S$_1$ surface elevations to coastal tide gauges (Sect. 4.4). Accordingly, results from all of our forward runs presented below have been inferred after completing the second model iteration.

### Internal Tide (IT) Drag Scheme

Consistent with previous studies of forward-modeled barotropic tides (Jayne and St Laurent 2001; Arbic et al. 2004; Egbert et al. 2004), surface elevations and tidal energies are poorly represented in DEBOT unless allowance is made for the substantial amount of drag generated by internal tides over major bathymetric features. With this proper dissipation mechanism omitted, area-weighted RMS differences $\overline{\Delta \zeta }$^1^ to the FES2012 reference tide ${\tilde{\zeta }}_R$ (complex sinusoid) are as large as 14.2 cm and 3.0 cm for M$_2$ and O$_1$, respectively. These values translate to a mere 72 and 79 % of sea surface height variance explained. Moreover, S$_1$ charts deduced from IT-free simulations display a number of apparent regional artifacts, such as persistently high amplitudes of the tide in the northern Atlantic ($\sim$1 cm) or the South China Sea ($\sim$2 cm) that have no correspondence in both the altimetric and hydrodynamic S$_1$ solutions of Ray and Egbert (2004). Parts of the OAM discrepancy of our initial control run (Table 7) to Ray and Egbert (2004)’s benchmark values can be understood in this light.

To increase the fidelity of our model tides and in particular S$_1$, we implemented the linear tidal conversion formulation of Zaron and Egbert (2006) as described and slightly modified by Green and Nycander (2013). In this parameterization, the local drag coefficient is explicitly proportional to the slope of the scattering topography

9 $$\begin{aligned} C_{IT} = \Gamma H \left( \nabla H\right) ^2\frac{N_b \overline{N}}{8 \pi ^2 \omega } \end{aligned}$$

where $\Gamma =50$ is a non-dimensional constant, $\omega$ denotes the frequency of the tidal motion, and theoretical buoyancy frequencies *N* follow from the prescription of a horizontally uniform abyssal stratification. Values of *N* at the ocean bottom ($N_b$) as well as vertical averages ($\overline{N}$) over the entire water column are calculated from Green and Nycander (2013)

10 $$\begin{aligned} N_b&= N_0 \mathrm {e}^{-H/1300} \end{aligned}$$

11 $$\begin{aligned} \overline{N}&= 1300 N_0 \left( 1 - \mathrm {e}^{-H/1300} \right) \frac{1}{H} \end{aligned}$$

with $N_0 = 5.24\cdot 10^{-3}$ s$^{-1}$, and *H* is the resting water depth (in m). Equation (9) is similar in form to the drag coefficient of Jayne and St Laurent (2001) and likewise ignores the influence of critical turning latitudes (where $\omega = f$, the Coriolis parameter) on the internal wave propagation characteristics. Yet, through scaling by $\omega$, the scheme is still frequency-dependent and thus applicable to only one specified constituent or, less strictly, to a particular tidal species. Considering our emphasis on the diurnal band, we fixed $\omega$ to $\Omega$, though that choice was found to improve the elevation accuracy of semidiurnal fringes (M$_2$) as well.

The IT drag formulation of Zaron and Egbert (2006) rather relies on scaling arguments than on a solid theoretical description of the topographically induced energy flux and thus contains a free parameter ($\Gamma$) to optimize the performance of the scheme. For practical reasons, we set $\Gamma =50$ (Green and Nycander 2013) and applied a secondary independent multiplier $\gamma$ at the order of $\mathcal {O}(1)$. With S$_1\left( p\right)$ taken from Ray and Egbert (2004), we could choose $\gamma$ in such a way that our model emulates the OAM values of this reference study. Alternatively, given the resemblance of S$_1$ to the global character of K$_1$ and O$_1$, the RMS misfit of forward-modeled diurnal gravitational constituents to altimetry-constrained solutions can be optimized. Both criteria do not lead to fully rigorous tuning experiments, as ocean dynamics vary from one tide to the other and allowance must be made for subtle differences of our time-stepping model with respect to Ray and Egbert (2004). In two separate suites of 40-day simulations with forcing specified for either $\{\mathrm {M}_2,\mathrm {O}_1,\mathrm {S}_1\}$ or the purely gravitational combination of $\{\mathrm {M}_2,\mathrm {O}_1,\mathrm {K}_1\}$, $\gamma$ was varied in steps of 0.5 within a range of 0.5–4. Tuning by RMS differences of K$_1$ and O$_1$ to the observed tide favored $\gamma = 1.5$, whereas the best match with Ray and Egbert (2004) in terms of OAM was achieved by $\gamma$ values in the vicinity of 3, although the eventual prograde annual nutation results appeared to be only weakly dependent on the exact value of $\gamma$ (within about 10 $\upmu$as). As a trade-off, we adopted $\gamma = 2$ as a “best estimate” for all of our S$_1$ runs below. RMS discrepancies to FES2012 produced by this setting are 5.6 cm (M$_2$), 1.7 cm (O$_1$), and 2.3 cm (K$_1$), implying more than 93 % of sea surface height variance explained for each tide; cf. similar statistics obtained by Arbic et al. (2004) with their ${1}/{2}^\circ$ barotropic model.

**Table 8:** Global angular momentum integrals of the S$_1$ ocean tide deduced from numerical modeling with varying pressure forcing climatologies^a^

	Ray/Egbert	Control^b^	MERRA	CFSR	ERA	EC-OP
*Mass*						
*x*	0.82 (158$^\circ$)	1.01 (163$^\circ$)	0.71 (199$^\circ$)	1.48 (149$^\circ$)	1.62 (161$^\circ$)	1.23 (163$^\circ$)
*y*	2.90 (306$^\circ$)	2.83 (298$^\circ$)	3.23 (321$^\circ$)	3.50 (319$^\circ$)	2.04 (295$^\circ$)	2.88 (304$^\circ$)
*z*	2.42 (219$^\circ$)	2.29 (207$^\circ$)	3.00 (239$^\circ$)	3.44 (248$^\circ$)	2.02 (234$^\circ$)	2.23 (218$^\circ$)
*Motion*						
*x*	1.72 (14$^\circ$)	1.60 (3$^\circ$)	1.84 (12$^\circ$)	1.46 (6$^\circ$)	1.26 (312$^\circ$)	1.54 (342$^\circ$)
*y*	1.62 (226$^\circ$)	1.53 (218$^\circ$)	1.69 (222$^\circ$)	1.18 (209$^\circ$)	1.58 (176$^\circ$)	1.76 (206$^\circ$)
*z*	2.57 (271$^\circ$)	2.65 (279$^\circ$)	1.66 (279$^\circ$)	2.22 (262$^\circ$)	3.43 (288$^\circ$)	2.71 (291$^\circ$)

^a^ Air pressure tides S$_1\left( p\right)$ for MERRA, ERA, and EC-OP are averages over 2004–2013, while the CFSR run draws on a 2004–2010 average. For brevity, the additional MERRA solution computed for the reduced 2004–2010 window is not tabulated but given in terms of nutation in Table 10. Amplitudes are in units of $10^{23}$ kg m$^2$ s$^{-1}$ and cotidal phases are given relative to Greenwich noon

^b^S$_1\left( p\right)$ from Ray and Egbert (2004) was deployed for the control run to validate our hydrodynamic model configuration including the tidal conversion scheme

S$_1$ amplitude and phase charts in our updated control runs with IT drag included are nearly indistinguishable from Fig. 3 of Ray and Egbert (2004), with previously noted regional anomalies (South China Sea, North Atlantic) eliminated (not shown). Table 8 underlines this sound agreement on the level of OAM values; cf. also the marked improvement with respect to our original, drag-free solution in Table 7. For both mass and motion components, phases differ by less than 15$^\circ$ throughout and deviations in amplitude are within $0.2\cdot 10^{23}$ kg m$^2$ s$^{-1}$. We calculated the corresponding contributions to the prograde annual nutation by aid of a standard protocol noted below, obtaining $21.9+i46.4$ $\upmu$as as a credible reproduction of the S$_1$ excitation value $20.7+i54.8$ $\upmu$as implied by the OAM terms of Ray and Egbert (2004); see Table 10. A moderate underestimation of the op component in DEBOT ($\sim$8 $\upmu$as) likely relates to differences in bathymetry or the treatment of ice shelves. Note also that the IT drag has no correspondence in the shallow water dynamics formulated by Ray and Egbert (2004), although a pertinent parameterization of tidal conversion, incorporated to the very same numerical model by Egbert et al. (2004), might have gone unmentioned.

Whether our prescription of topographically generated drag at the S$_1$ frequency is physically justified or not is a potentially interesting issue but not of immediate importance for the topic in hand. One of the vexing problems related to this question is that our forward model operates in the time domain, while internal waves are preferably studied in the frequency domain. Here, we have adopted a diagnostic approach, inferring the need for additional mid-ocean dissipation by comparing our initial results for S$_1$ and other diurnal tides to established reference charts. On a side note, also the discrepancies to coastal tide gauge estimates of S$_1$ (next section) are markedly lower when internal wave drag is parameterized.

### Hydrodynamic Solutions and Validation with Tide Gauge Data

**Fig. 6:**
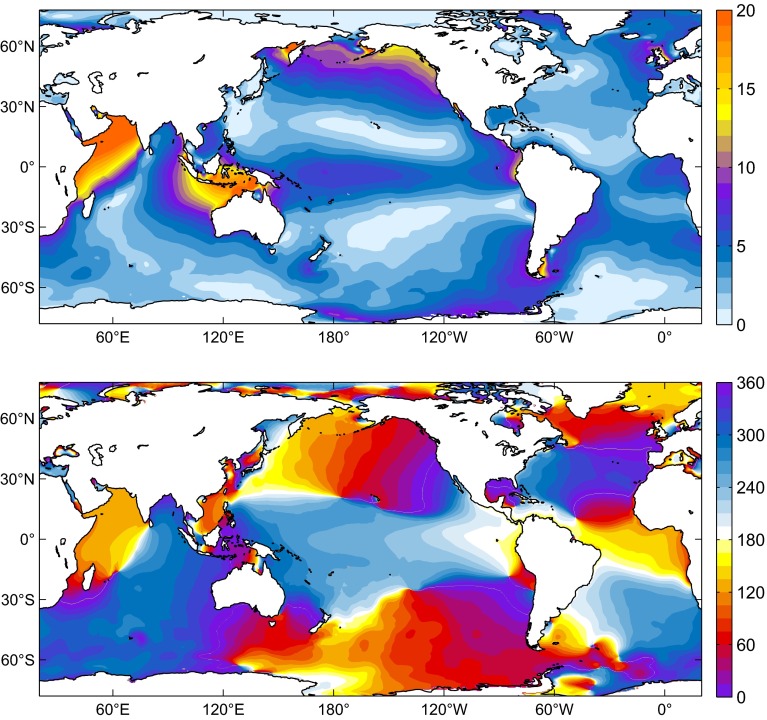
Amplitudes (*top*, in mm) and Greenwich phase lags (*bottom*, in deg) for the sea level signal due to forcing by the S$_1$ atmospheric pressure tide from MERRA (2004–2013 average). Cotidal phases are relative to Greenwich noon

**Fig. 7:**
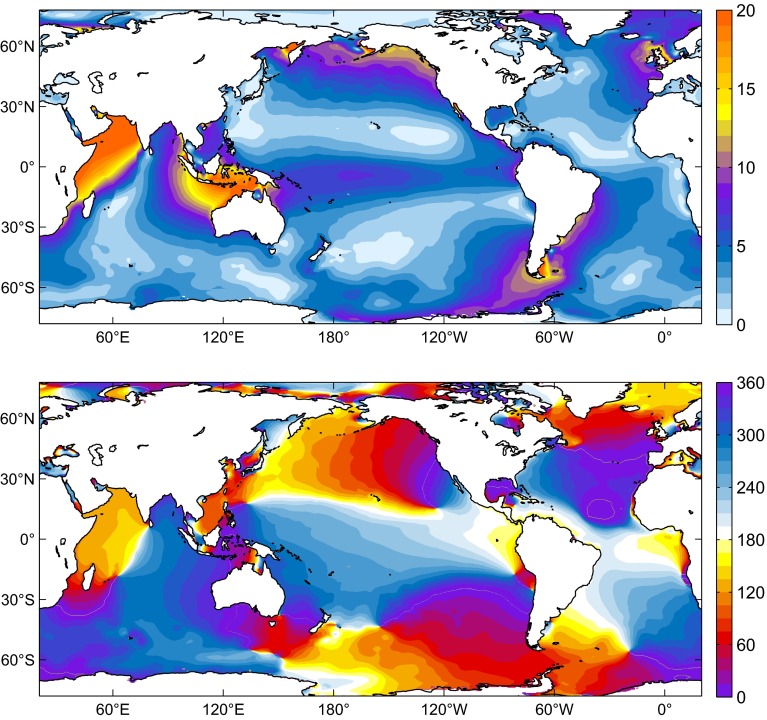
Same as Fig. 6 but for S$_1\left( p\right)$ from CFSR (2004–2010 average)

**Fig. 8:**
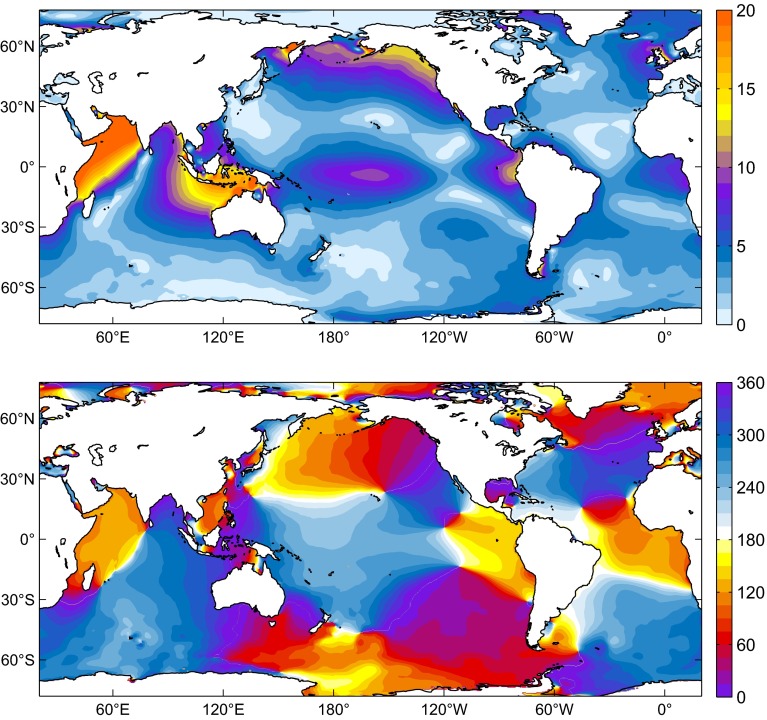
Same as Fig. 6 but for S$_1\left( p\right)$ from EC-OP (2004–2013 average)

Tidal elevation charts obtained from numerical modeling are shown in Figs. 6, 7 and 8 for MERRA, CFSR, and EC-OP, while ERA has been left aside as the solution with probably the least accurate forcing data; cf. Sect. 3.3.

Similarities with published S$_1$ charts (e.g., Dobslaw and Thomas 2005; Ponte and Vinogradov 2007) are readily apparent and particularly striking for our EC-OP model as compared to the S$_1$ tide of Ray and Egbert (2004), who also employed ECMWF operational analysis data. Measured against Fig. 4 of these authors, DEBOT appears to underestimate the tide in Baffin Bay and the Sea of Okhotsk, probably due to differences in seafloor topography or the specification of dissipative processes. Such small-scale deficiencies in high latitudes are, however, of little relevance for the global OAM integrals.

All computed S$_1$ realizations agree in terms of the global character of the tide, but basin-wide features can vary substantially in response to different pressure forcing data. Specifically, the secondary peaks of S$_1\left( p\right)$ around 60$^\circ$S in the CFSR climatology (Fig. 2) induce sea level signals in the Southern Ocean that exceed the corresponding tidal variability from MERRA and EC-OP by about 1–5 mm. Amplitudes in the North Atlantic are also comparatively high in the CFSR solution, whereas EC-OP displays the largest S$_1$ tide in the Tropical Pacific, consistent with the overestimation of equatorial pressure gradients as exposed by Fig. 5c.

**Fig. 9:**
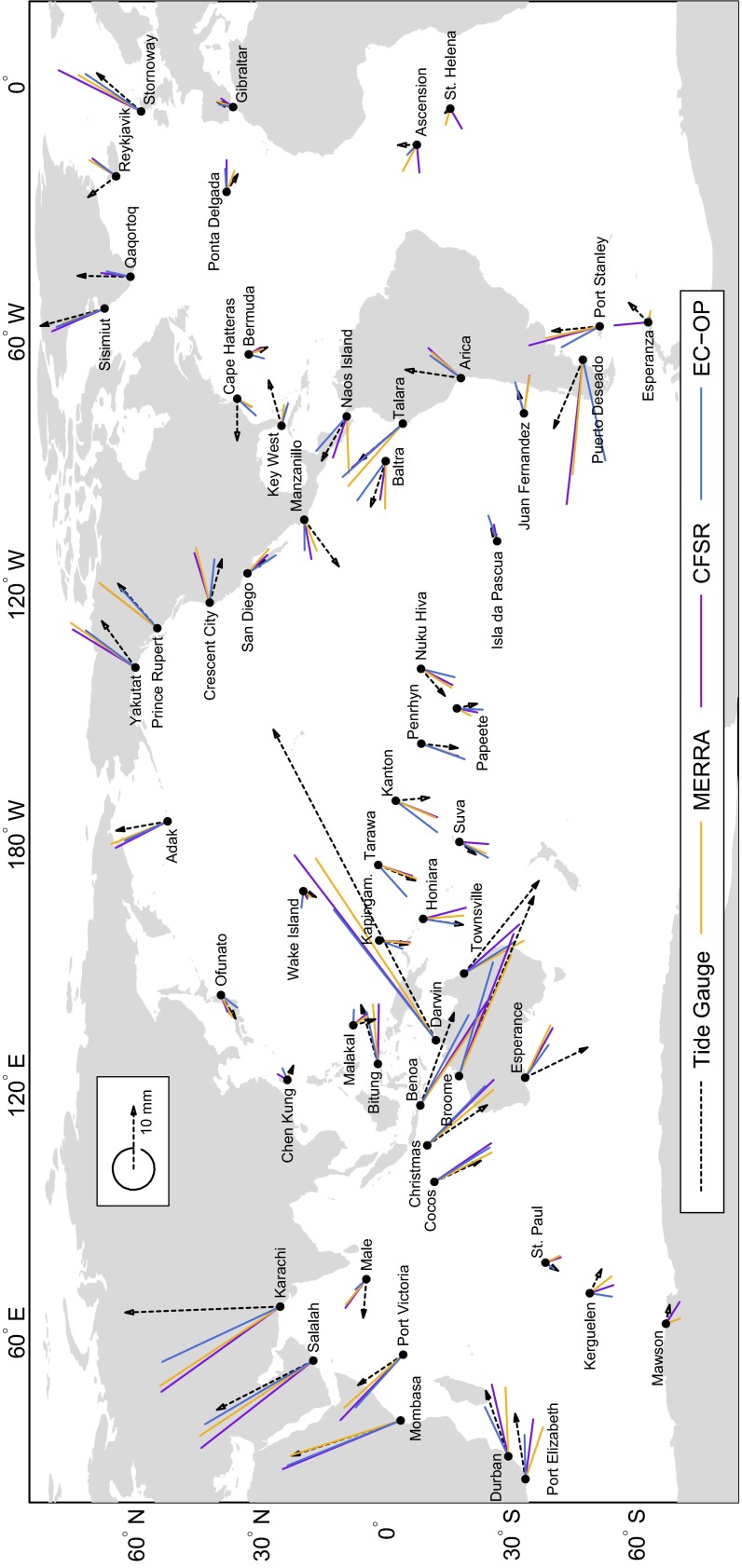
Observed and simulated S$_1$ sea level signals at 56 tide gauge locations. Greenwich phase lags follow the Doodson convention for radiational tides (Eq. 12) and are reckoned counterclockwise. Tide gauge estimates (*black* phasors), taken from Ponchaut et al. (2001) and Ray and Egbert (2004), have been corrected for the influence of the small gravitational S$_1$ tide. Results from ERA are not shown for display purposes

Empirical knowledge of S$_1$ to validate our forward simulations comes from globally distributed coastal tide gauges. Such a point-wise verification may imply little with regard to Earth rotation, yet it is instructive to determine whether individual models systematically perform better than others. Harmonic estimates of S$_1$ at some 200 places are available in the online datasets of Ponchaut et al. (2001)^2^, who tidally analyzed multi-year time series of hourly sea level records assembled both by BODC (British Oceanographic Data Centre) and UHSLC (University of Hawaii Sea Level Center). We extracted a subset of 51 estimates from Ponchaut’s compilation, excluding sites where the tide is effectively zero (Hawaii, Japan, Maldives, Central Atlantic) or all model predictions are equivocally different from the observations, e.g., due to unresolved coastal geometries (Prudhoe Bay, Bluff Harbour). Moreover, in order to avoid biases toward densely sampled regions, only a few locations were retained in the equatorial Pacific and further thinning was applied to higher-amplitude S$_1$ estimates in close proximity to each other (Arabian Sea, Gulf of Alaska). Our final 56-station set also includes five tide gauges from Ray and Egbert (2004) (Karachi, Benoa, Broome, Bermuda, Gibraltar) and is presented in Fig. 9. Valuable additions to this network, e.g., in the seas of Southeast Asia or along the coast of Brazil were pinpointed in the PSMSL (Permanent Service for Mean Sea Level) holdings, yet we refrained from a thorough tidal analysis of these hourly data in the frame of the present work.

The collected harmonics are aggregate measures of both the radiational S$_1$ ocean tide and the much smaller gravitationally driven component, S$_1^g$. The latter must be removed from the in situ data to rigorously compare with our numerical solutions of S$_1$ that are solely forced by atmospheric pressure. To that end, we evaluated the S$_1^g$ chart given in Appendix “The Gravitational S_1_ Ocean Tide” at the locations of our 56 gauges and changed the phase reference from the tide-generating potential to the simple radiational S$_1$ argument; see Ray and Egbert (2004) for details. Tidal components after subtraction of the gravitational signal (usually 1–3 mm) are displayed as phasors in Fig. 9 and cover an amplitude range from 56 mm at Darwin (10-year mean estimate) down to 1.3 mm at St. Helena (4-year mean). Confidence intervals for all small-magnitude S$_1$ determinations from only a few years of data appear to be sufficiently tight in the analysis of Ponchaut et al. (2001) to warrant the inclusion of these stations in our network. The median time series length over all 56 tide gauges is 8 years.

Simulated S$_1$ signals at each gauge location were taken from the nearest pelagic point in our ${1}/{3}^\circ$ model and are illustrated for MERRA, CFSR, and EC-OP in Fig. 9. In general, all hydrodynamic solutions agree reasonably well with the observations, even though disparities on the order of a few mm must be accepted at most sites. The unusually large tide at Darwin (56.0 mm after reduction of the gravitational signal) has been addressed by Ray and Egbert (2004) and appears to be a very local modulation of S$_1$ in a shallow (5-m) inlet that is approximated by a coarse gridpoint of 10 m depth in our bathymetry. MERRA and CFSR amplitudes at Darwin are 35 mm and 37 mm and thus somewhat closer to the observation than the model estimate of Ray and Egbert (2004). In a broader context, the collection of tide gauges across the Atlantic testify to the shortcomings of the CFSR solution, evident, e.g., from the amplitude excess at Puerto Deseado, Port Stanley, and Esperanza. Moreover, most of the EC-OP estimates in the equatorial Pacific are too high, implying that this model must be treated with caution for studies of axial changes in Earth’s rotation.

We have also attempted to express the varying accuracies of our hydrodynamic solutions by global statistical measures in Table 9. Median absolute differences as well as RMS misfits, given both as absolute and amplitude-normalized values, show little variations among the four models if all 56 tide gauge locations are considered. This result conforms with Fig. 9 inasmuch as the simulated tide at larger-amplitude sites tends to differ from the observation in the same way for all models; see, e.g., Karachi, Port Victoria, Yakutat, or all Australian stations. Somewhat more instructive statistics are obtained if the network is limited to stations below a certain amplitude threshold. Table 9 specifies results for an 8-mm threshold that preserves 31 tide gauges, most of them being located at mid-ocean islands. In this variant, MERRA outperforms all other models both in terms of MAD and RMS values, of which the reduction from 1.9 mm to 1.6 mm is significant at the 0.15 level. Results for the second MERRA run associated with the 2004–2010 pressure tide average (not shown) are slightly inferior, comparable with the MAD statistics of the two ECMWF models. Note also that the ERA tide, for which we have made reservations with regard to the forcing data (Sect. 3.3), is among the best models in Table 9 owing to a particularly good match with tide gauge estimates in the North Pacific.

**Table 9:** RMS misfits and median absolute differences (both in mm) of simulated tidal harmonics with 56 tide gauge estimates from Ponchaut et al. (2001) and Ray and Egbert (2004)

	All stations		Amplitude $<8$ mm^a^	
	RMS^b^	MAD	RMS^b^	MAD
MERRA	2.6 (0.34)	2.1	1.6 (0.38)	1.3
CFSR	2.8 (0.40)	2.2	1.9 (0.49)	1.9
ERA	2.7 (0.35)	1.9	1.9 (0.43)	1.5
EC-OP	2.8 (0.37)	2.2	1.9 (0.45)	1.6

^a^Of 56 stations, 31 feature in situ amplitudes less than 8 mm

^b^Numbers in parentheses are amplitude-normalized RMS differences

### Contribution of the Oceanic S$_1$ Tide to Nutation

Global OAM integrals derived from our numerical modeling efforts are compiled in Table 8 and exhibit a considerable scatter in accordance with the large-scale inter-model differences noted in the previous section. There is, however, a broad consensus that the *x* and *y* mass terms, i.e., the two single most important components with regard to nutation, are in the order of $\sim$1.0 $10^{23}$ kg m$^2$ s$^{-1}$ (160$^\circ$ phase lag) and $\sim$3.0 $10^{23}$ kg m$^2$ s$^{-1}$ (0$^\circ$ phase lag), respectively. The tabulated harmonics were translated to nutation values in essentially the same manner as AAM through multiplication of mass and motion terms with the proper transfer function coefficients $\tilde{T}^{p,w}\left( \sigma \right)$; cf. Eqs. (1) and (2). As this scheme is initialized by a demodulation of angular momentum series in the time domain, we first discretized *x* and *y* OAM components over a 3-year window by a cosine function using the Doodson argument for the radiational S$_1$ tide (see Appendix A of Ray and Egbert 2004)

12 $$\begin{aligned} T+180^\circ -\varphi _H \end{aligned}$$

where *T* is Universal Time and $\varphi _H$ are respective phase lag values as given in Table 8. Demodulated equatorial OAM series were then cleansed from non-seasonal signals (that is, the S$_1$ contribution to prograde polar motion), fitted to the periodic forcing model (Eq. 4), and expressed as nutation harmonics through Eq. (1) with phases referred to the fundamental arguments of gravitational diurnal tides.

**Table 10:** Periodic oceanic contributions to the prograde annual nutation ($\upmu$as) as inferred from the model-specific OAM values of Table 8 ^a^

	Mass		Motion		Total	
	ip	op	ip	op	ip	op
Ray/Egbert	26.2	54.7	$-5.5$	0.1	20.7	54.8
Control	27.1	45.4	$-5.2$	1.0	21.9	46.4
MERRA	19.7	59.8	$-5.8$	0.4	13.9	60.2
MERRA^b^	27.9	65.7	$-6.4$	0.3	21.5	66.0
CFSR^b^	6.1	83.9	$-4.1$	0.8	2.0	84.7
ERA	−0.1	34.1	$-2.5$	4.3	−2.6	38.4
EC-OP	18.0	51.5	$-5.0$	2.8	13.0	54.3

^a^Results are split up into the contributions from tidal heights (mass term) and currents (motion term). In- and out-of-phase components are referred to the fundamental arguments of nutation (Table 4) and the sign convention is that of Koot and de Viron (2011)

^b^Forced by the respective pressure tide average from 2004–2010

Table 10 summarizes the various estimates of the oceanic S$_1$ effect in nutation. Formal errors have been omitted as they are effectively zero given our usage of perfect sinusoids for the angular momentum time series. Overall, the nutation results from all model runs are reasonably consistent, ranging from 0 to 20 $\upmu$as in the ip terms and roughly 40 to 60 $\upmu$as for the op component, with 90 % of the signal coming from the mass component. MERRA (2004–2013 average) and EC-OP produce a particularly close match within 6 $\upmu$as, and the moderate increase in magnitude for the reduced MERRA time span (2004–2010) is in fact expected on grounds of the time-variable amplitudes of S$_1\left( p\right)$ (Fig. 5). For CFSR, the large-scale enhancement of tidal heights in the Indian Ocean and the North Atlantic (Fig. 7) combine to yield an excessive op estimate of 84.7 $\upmu$as. Nonetheless, the spread of nutation values is significantly smaller than that of previous inter-model comparisons, conducted, e.g., by Brzeziński (2008) based on IB-corrected OAM values from much coarser (>1°) barotropic and baroclinic models. We have also mapped the fine-resolution S$_1$ tide of FES2012 to the prograde annual band, finding a nutation estimate of $-2.1+i49.1$ $\upmu$as that roughly matches our ERA harmonic. This agreement likely relates to similarities in the barometric forcing data, as the hydrodynamic core of FES2012 includes pressure loading from the 3-h ECMWF delayed cutoff stream (Carrère et al. 2012). Prograde annual nutation estimates for FES2012 as well as the model of Ray and Egbert (2004) are also tabulated in Schindelegger et al. (2015), albeit with an internal conversion error at the order of 10 $\upmu$as which has been corrected in the frame of the present study.

## Comparison with Geodetic Observations

At an amplitude of $\sim$25.6 mas, the prograde annual nutation is among the principal signal components in Earth’s celestial motion and driven almost exclusively by the action of the solar gravitational torque on the equatorial bulge. In the MHB theory, the term is modulated to a minor degree by anelasticity ($-10-i4$ $\upmu$as), electromagnetic torques ($-14+i6$ $\upmu$as for both core mantle and inner core boundaries), geodesic nutation ($-30+i0$ $\upmu$as), and the angular momentum exchange of the solid Earth with the gravitational ocean tide, S$_1^g$ ($-21+i22$ $\upmu$as); see also Table 2 of Brzeziński et al. (2004). With these contributions accounted for, theory and observation of the prograde annual nutation produce a mismatch of $-10.4+i108.2$ $\upmu$as that has been attributed by MHB to the thermal atmospheric S$_1$ tide and, implicitly, to the radiational S$_1$ tide in the ocean.

Realizations of the very same residual have been also derived by Koot et al. (2010) in the frame of a time domain Bayesian inversion of nutation observations including non-linearities and additional terms in the functional model. Koot et al. (2010) used 10 years of additional VLBI data compared to MHB but employed identical corrections for geodesic nutation and the gravitational ocean tide. It is thus not surprising that the empirical S$_1$ estimate of these authors is numerically very similar to the MHB residual; from a joint inversion of three nutation series from different analysis centers Koot et al. (2010) deduced a harmonic of $0+i107$ $\upmu$as. Corresponding SD in both ip and op components are 4 $\upmu$as but probably underestimated and arguably better represented by the single-solution error of 7 $\upmu$as; cf. Table 1 of Herring et al. (2002).

Residual VLBI-based nutations obtained after reduction of known effects do not necessarily provide a clean account of the rotational signal associated with the global S$_1$ tide. Both unconsidered Sun-synchronous effects as well as inaccuracies in the incorporated relativistic or geophysical corrections at the prograde annual frequency might perturb empirical S$_1$ estimates. However, theoretical values of geodesic nutation are known to great precision (Fukushima 1991), whereas anelastic and electromagnetic coupling contributions to the S$_1$ band are too small ($<20$ $\upmu$as) to leave room for significant changes even if the MHB treatment of these effects is revised. The contribution from the gravitational ocean tide is somewhat larger (see above) and in fact subject to uncertainties owing to the manner in which it has been included in the nutation formalism. In detail, MHB inferred a harmonic of $-21+i22$ $\upmu$as from OAM estimates of K$_1$, P$_1$, O$_1$, and Q$_1$ (Chao et al. 1996) via scaling relationships that were optimized for the diurnal band on a broad scale instead of particular tidal lines. We therefore recomputed the effect based on S$_1^g$ OAM integrals deduced in Appendix “The Gravitational S_1_ Ocean Tide” (Table 12), applying essentially the same time domain discretization as in Sect. 4.5 but with phases referred to the present-day argument of the gravitational S$_1$ tide, that is, $T+295.66^\circ +90^\circ$ (Ray and Egbert 2004). Multiplication of adjusted mass and motion term coefficients (Eq. 4) with the respective transfer ratios (Eq. 1) yielded a harmonic of $-15.2+i16.8$ $\upmu$as. This value is about 5 $\upmu$as smaller than the intrinsic MHB estimate in both ip and op components, and a similar decrement is assumed for the analysis of Koot et al. (2010), who also utilized OAM data of Chao et al. (1996). The corresponding correction was imposed on the prograde annual nutation residuals of both studies, resulting in the empirical S$_1$ terms given in Table 11.

**Table 11:** Estimates of the prograde annual nutation ($\upmu$as) as driven by the global radiational S$_1$ tide in the coupled atmosphere–ocean system^a^

	In-phase	Out-of-phase
MERRA	$-8.0$	106.0
MERRA^b^	$-6.1$	115.2
CFSR^b^	$-32.3$	172.3
ERA	$-38.7$	95.9
EC-OP	$-9.4$	121.8
Brzeziński et al. (2004)	113.1	96.1
Brzeziński (2011)	$-60.6$	83.9
VLBI (MHB)	$-16.2$	113.4
VLBI Koot and de Viron (2011)	$-5.8$	112.2
1$\sigma$ error	7	7

^a^MERRA, CFSR, ERA, and EC-OP results are superpositions of the harmonics from Tables 5 and 10. For comparison, earlier estimates from Brzeziński et al. (2004) and Brzeziński (2011) are also shown and have been multiplied by $-1$ to account for differences in the definition of nutation amplitudes. VLBI values, with formal errors taken from Herring et al. (2002), have been cleared of the gravitational S$_1$ tide influence by using the results of Appendix “The Gravitational S_1_ Ocean Tide”; see the text for further details

^b^Forced by the respective pressure tide average from 2004–2010

Additional regard must be paid to the distortion of observed nutations through Sun-synchronous thermal deformations of some or all VLBI telescopes (Herring et al. 1991). This effect is now rigorously accounted for in VLBI analyses by means of a conventional procedure using on-site values of temperature, but the matter of discussion is whether a proper deformation correction was employed in the computation of nutation series that underlie the studies of MHB as well as Koot et al. (2010). Here, we draw on different evidences to argue that the effect was sufficiently well modeled, e.g., by early reduction schemes similar to Sovers et al. (1998) (Sect. G, *ibid.*).

Single-session runs with our in-house VLBI software (Böhm et al. 2012) showed that a spurious prograde annual nutation variability of about 20 $\upmu$as in both ip and op components is incurred by analyses that explicitly omit corrections for solar heating of VLBI antennas. Hence, a persistent bias of $\sim$30 $\upmu$as should be evident in the comparison of nutation series from present-day VLBI analyses with the MHB model, assuming that for the latter diurnal deformation signals were neglected. This comparison is actually realized in the form of the IERS CPO data given w.r.t. the MHB series, of which a windowed Fourier analysis in the prograde annual band has already been presented in Fig. 1. The absence of any systematic distortion at the order of 30 $\upmu$as is readily apparent, in particular in the post-2000 period that features little uncertainty in the CPO estimates. Moreover, Table 3 of Koot et al. (2010) itself implies a proper modeling of solar heating in previous VLBI solutions. Among the three nutation series inverted by these authors, a thermal deformation correction is unambiguously identified for the IAA (Institute of Applied Astronomy, Moscow) data; see the corresponding documentation available at ftp://ivsopar.obspm.fr/vlbi/ivsproducts/eops/ (accessed 14 October 2015). The treatment of the heating effect is undisclosed in the description of the other two series but, reassuringly, the associated prograde annual nutation residuals are not systematically offset from the IAA solution. Note also that the MHB estimate blends in well with Koot et al. (2010)’s results for various analysis centers. Artificial nutation signals related to antenna structure changes can be therefore deemed insignificant and the collected VLBI-based empirical S$_1$ terms should be accurate enough to serve as reference values for our geophysical model estimates.

**Fig. 10:**
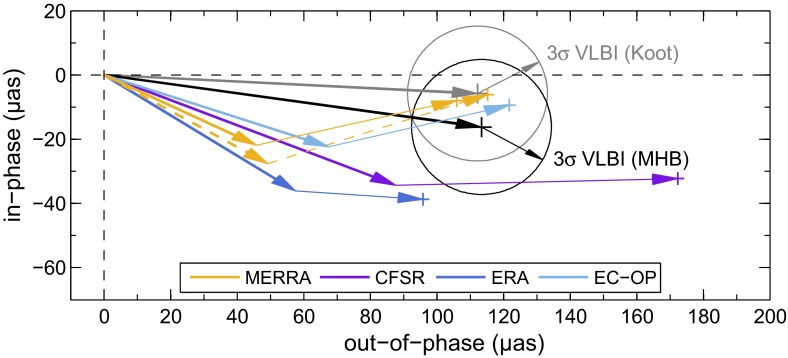
Atmosphere–ocean contributions to the prograde annual nutation as obtained from four atmospheric (re)analyses and the DEBOT time-stepping model forced by respective air pressure tide climatologies. The excitation values are multi-year averages either from 2004 to 2010 (CFSR and MERRA dashed *orange* lines) or from 2004 to 2013 (ERA, EC-OP, and MERRA solid *orange* lines), where the bold phasor of each model represents the atmospheric contribution (Table 5) and the superimposed thin phasors have been computed from OAM values (Table 10). The VLBI-based prograde annual nutation residuals of MHB and Koot et al. (2010) in Table 11 are shown in *black* and *gray*, respectively, equipped with an error ellipse of radius 21 $\upmu$as that corresponds to the threefold VLBI SD in the prograde annual band

This excitation balance is elaborated in Table 11 as well as in Fig. 10

and represents the core result of our study. Both MERRA (either solution) and EC-OP estimates agree with geodetic observations of the prograde annual nutation at the 10 $\upmu$as level, well below the threefold SD of the VLBI solutions. A discrepancy of only 3 $\upmu$as is found between MERRA (2004–2010) and the joint inversion residual of Koot et al. (2010), even though such a close fit might be fortuitous considering the time variability of S$_1$ excitation terms and the uncertainties of the involved numerical models. By and large, the consistency of MERRA with VLBI data is in keeping with its good performance in comparison with atmospheric and oceanic ground truth data. Further correlations between model-specific in situ statistics and the nutation results in Fig. 10 are less obvious, but a closer inspection of tide gauge estimates in Durban, Port Elizabeth, Esperance, and Mawson (Fig. 9) revealed that ERA systematically underestimates the ocean tide in the South Indian Ocean, by about 2 mm compared to the observed sea level signal and to other simulations (MERRA, EC-OP). We implemented a split-up of the retrograde OAM mass term into contributions from different basins, showing that the broadscale S$_1$ features in the South Indian Ocean have indeed a significant bearing on the op component of the ocean-driven prograde annual nutation. The 30-$\upmu$as deviation of the ERA model from, e.g., MHB’s nutation estimate is thus attributed in large part to a regional signal loss in terms of tidal elevation in the southern hemisphere.

Table 11 also places our final results in the context of previous geophysical modeling efforts by Brzeziński et al. (2004) and Brzeziński (2011). In these studies, a balance with VLBI observations has been mostly impeded by deficiencies in the ip component of the modeled S$_1$ excitation. To some extent, the choice of meteorological data (NCEP R1, ERA) is critical in either investigation, and further errors relate to insufficiencies of the utilized ocean models regarding the simulation of the S$_1$ tide. Specifically, the model deployed by Brzeziński (2011) has been optimized for a range of timescales and full baroclinic variability, for which coarse horizontal resolutions (1.875$^\circ$) greatly reduce computational costs. We tested the impact of a 1$^\circ$ discretization in DEBOT, obtaining somewhat anomalous S$_1$ charts and increasingly negative ip components of the ocean-driven prograde annual nutation ($-15$ $\upmu$as decrement). Brzeziński et al. (2004) analyzed the output of a barotropic model with a comparably coarse domain representation (1.125$^\circ$ spacing) but also with SAL dynamics neglected. This omission alters nutation amplitudes by roughly 30 $\upmu$as, and perturbations of similar size occur if dissipative processes are imperfectly accounted for. Such shortcomings have been redressed in the present work, by drawing on modern insights into the forward modeling of global ocean tides.

## Concluding Discussion

S$_1$ tidal excitations of nutation in the order of 3.5 mm at the Earth’s surface ($\sim$120 $\upmu$as) have constituted an anomaly to non-rigid nutation theories for decades. We have put forth an explanation of geodetic observations of the effect based on reanalysis data from MERRA and operational ECMWF analysis fields, complemented by numerical hydrodynamic solutions for the radiational S$_1$ tide in the ocean. Atmospheric contributions averaged over 2004–2013 are $-21.9+i45.8$ $\upmu$as (MERRA) and $-22.4+i67.5$ $\upmu$as (EC-OP) and combine well with the respective oceanic estimates ($13.9+i60.2$ $\upmu$as, $13.0+i54.3$ $\upmu$as) to match the VLBI-observed S$_1$ terms within 10 $\upmu$as. No attempt was made to rigorously quantify the uncertainty of these geophysical model estimates, but we suppose that the errors are comparable to the threefold VLBI SD in the prograde annual band (21 $\upmu$as). In particular, the atmospheric mass term is among the least robust components of the global S$_1$ excitation given its dependence on the small second-order tesseral surface pressure wave. Table 5 documents an inter-model spread of 25 $\upmu$as (excepting CFSR) for the pressure-driven nutation, comprising also uncertainties due to inter-annual S$_1$ variations that have not been completely removed by the chosen 10-year averaging window. In contrast, our forward simulations of the radiational ocean tide should be fairly reliable on condition that the barometric forcing data themselves are accurate. Only weak ($<10$ $\upmu$as) and possibly counterbalancing influences of bathymetry and drag parameterization have been noted in Sect. 4. We also conducted DEBOT runs on C-grids finer than ${1}/{3}^\circ$, obtaining nutation harmonics of only a few $\upmu$as deviation with respect to the estimates given in Table 10.

Differences in the diurnal cycle of modern atmospheric assimilation systems have played one of the recurring themes throughout this paper and are not necessarily smaller than the high-frequency disparities among earlier generation reanalyses; recall, e.g., our assessment of the CFSR pressure data. A more coherent representation of air tides is evidently tied to a near-global observing system with continuous sub-daily sampling (Schindelegger and Dobslaw 2016) but also depends on other aspects of the (re)analysis framework. Poli et al. (2013) emphasized the importance of at least hourly radiation time steps—a condition that is met neither by CFSR nor by JRA-55 (Kobayashi et al. 2015), which was also examined in a preliminary stage of our study but led to deficient AAM/OAM phasors. Moreover, the formulation of the assimilation technique can have implications for tides, considering in particular that the variational analysis (3DVar or 4DVar; see Table 1) is usually performed in sequential 12-h windows without accounting for continuity of state variables at the transition epochs. The resulting perturbations occur at integer fractions of a solar day and potentially fold to an artificial S$_1$/S$_2$ variability. Such spurious signals are, however, minimized in the special case of MERRA through its Incremental Analysis Update method (Rienecker et al. 2008), which might ultimately figure into the good performance of MERRA throughout our study. Finally, the accuracy of S$_1$ in global analysis models is closely linked to the fidelity with which moist convection and latent heat flux can be simulated. Deficiencies in these quantities relate to imperfect physical paramaterizations or uncorrected biases in observations (Meynadier et al. 2010) and are, e.g., well documented for ERA (Dee et al. 2015).

Displaying little long-term variability both in the celestial pole offsets (Fig. 1) and in the atmospheric S$_1$ excitation, the 2004–2013 period has provided the ideal setting to study the mean harmonic atmosphere–ocean contribution to the prograde annual nutation. A reliable estimation of the temporal evolution of nutation amplitudes is still challenging, though. These signals differ substantially among the probed models and are masked by noise interferences as well as spurious variabilities when the frozen assimilation routines of reanalyses are confronted with new types and volumes of observations. Judging from Figs. 1, 3, and similar analyses in Bizouard et al. (1998), an upper bound of 30 $\upmu$as appears to be a plausible estimate for the irregular departures from a simple sinusoidal S$_1$ term in nutation. These vacillations dictate the likely accuracy of upcoming nutation models but also serve as an incentive for future foundational research, relating climate signals in geodetic observations with the time-variable excitation quantities from geophysical fluid models.

## Figures and Tables

**Fig. 11:**
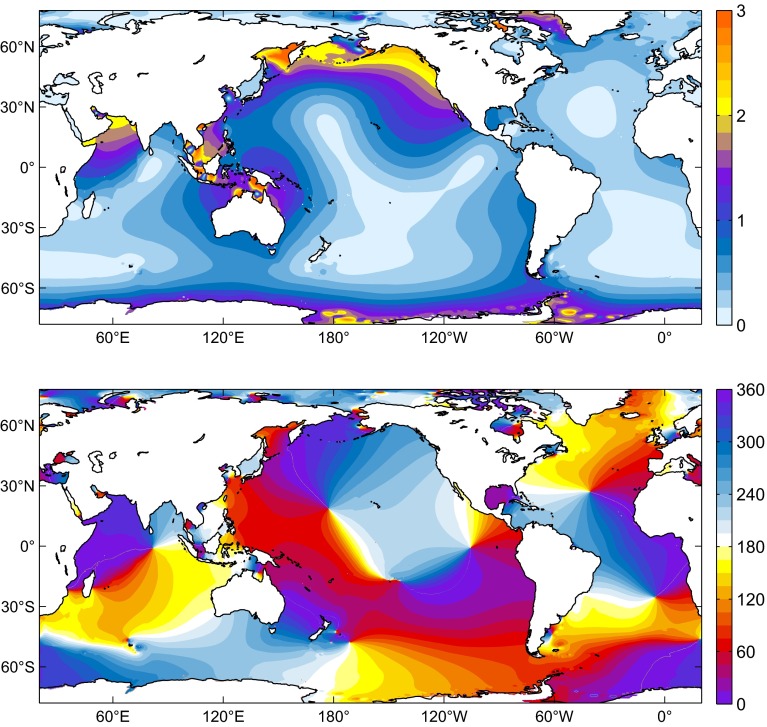
Cotidal elevation charts for the gravitational S$_1^g$ tide in the global ocean as computed from FES2012 solutions of K$_1$ and P$_1$. Amplitudes (in mm) are shown in the *upper panel*, Greenwich phase lags (°) in the *lower panel*

## Appendix 1: The Gravitational S$_1$ Ocean Tide

Following Sect. 2c of Ray and Egbert (2004), a modern-day chart of S$_1^g$ in the global ocean is readily computed from observations of the gravitational P$_1$ and K$_1$ tides, which are separated from the S$_1$ band by only 1 cpy. Tidal heights of K$_1$ were extracted from the FES2012 atlas on a ${1}/{16}^\circ$ mesh, moderately downsampled, and scaled to local S$_1^g$ amplitudes using a factor of 1.98/368.74, that is, the ratio of gravitational potentials at S$_1$ and K$_1$. Greenwich phase lags were calculated as averages from both the K$_1$ and P$_1$ charts and are illustrated together with the amplitudes of S$_1^g$ in Fig. 11. Associated barotropic currents follow from the velocity ($\mathbf {u}$) grids of K$_1$ and P$_1$ in the FES2012 model, based on the same interpolation procedures as for the local elevation. To serve Sect. 5, we have mapped heights and currents of S$_1^g$ to global OAM values as documented in Table 12. Note that these harmonics might be also derived from a direct application of admittance relationships to the angular momentum values of the K$_1$ and P$_1$ tides.

**Table 12:** OAM values of the gravitational S$_1^g$ tide, with amplitudes given in units of $10^{23}$ kg m$^2$ s$^{-1}$ and Greenwich phase lags referred to the tide-generating potential

	Mass	Motion
*x*	0.26 (306$^\circ$)	0.31 (288$^\circ$)
*y*	0.74 (223$^\circ$)	0.42 (193$^\circ$)
*z*	0.09 (357$^\circ$)	0.43 (128$^\circ$)

## Footnotes

1 Computed as $\overline{\Delta \zeta } = \sqrt{\frac{\iint \left| \tilde{\zeta } - \tilde{\zeta }_R\right| ^2 dA}{2\iint dA}}$, equivalent to the time-averaged expression of Arbic et al. (2004), with grid points poleward of 66$^\circ$ and waters shallower than 1000 m excluded.

2 http://www.bodc.ac.uk/projects/international/woce/tidal_constants/. Accessed 9 October 2015.
